# Chemical, Metabolic, and Cellular Characterization of a FtsZ Inhibitor Effective Against *Burkholderia cenocepacia*

**DOI:** 10.3389/fmicb.2020.00562

**Published:** 2020-04-07

**Authors:** Laurent R. Chiarelli, Viola Camilla Scoffone, Gabriele Trespidi, Giulia Barbieri, Olga Riabova, Natalia Monakhova, Alessio Porta, Giulia Manina, Giovanna Riccardi, Vadim Makarov, Silvia Buroni

**Affiliations:** ^1^Laboratory of Molecular Microbiology, Department of Biology and Biotechnology “L. Spallanzani”, University of Pavia, Pavia, Italy; ^2^Federal Research Centre “Fundamentals of Biotechnology” of the Russian Academy of Sciences, Moscow, Russia; ^3^Organic Chemistry Section, Department of Chemistry, University of Pavia, Pavia, Italy; ^4^Microbial Individuality and Infection Group, Cell Biology and Infection Department, Institut Pasteur, Paris, France

**Keywords:** *Burkholderia cenocepacia*, new antimicrobials, drug resistance, cell division, cystic fibrosis

## Abstract

There is an urgent need for new antimicrobials to treat the opportunistic Gram-negative *Burkholderia cenocepacia*, which represents a problematic challenge for cystic fibrosis patients. Recently, a benzothiadiazole derivative, C109, was shown to be effective against the infections caused by *B. cenocepacia* and other Gram-negative and-positive bacteria. C109 has a promising cellular target, the cell division protein FtsZ, and a recently developed PEGylated formulation make it an attractive molecule to counteract *Burkholderia* infections. However, the ability of efflux pumps to extrude it out of the cell represents a limitation for its use. Here, more than 50 derivatives of C109 were synthesized and tested against Gram-negative species and the Gram-positive *Staphylococcus aureus*. In addition, their activity was evaluated on the purified FtsZ protein. The chemical, metabolic and cellular stability of C109 has been assayed using different biological systems, including quantitative single-cell imaging. However, no further improvement on C109 was achieved, and the role of efflux in resistance was further confirmed. Also, a novel nitroreductase that can inactivate the compound was characterized, but it does not appear to play a role in natural resistance. All these data allowed a deep characterization of the compound, which will contribute to a further improvement of its properties.

## Introduction

Cystic fibrosis (CF) patients are continuously subjected to antibiotic therapies due to lung colonization both by Gram-positive and –negative bacteria. Among the latter, *Burkholderia cenocepacia* represents a main concern being responsible for the so-called “*cepacia* syndrome,” a necrotizing pneumonia which can lead to patient's death (Salsgiver et al., 2016). Indeed, this opportunistic pathogen is among the bacterial species CF patients should be worried about (Jones, 2019). Moreover, infections caused by *B. cenocepacia* are still a major contraindication to lung transplantation, although certain centers do admit infected patients in the list (Dupont, 2017). An important characteristic, which renders these bacteria particularly dangerous, is their resistance toward most antibiotics used in clinical practice (Scoffone et al., 2017). This limits the therapeutic options available to treat the infections, and there are no standardized set of antibiotics for treatment. In this context, new antibacterials are highly necessary, although their development may be limited by poor commercial interest in *Burkholderia* infections because they are rare, with approximately 2.6% of CF patients infected with these bacteria (https://www.cff.org/Research/Researcher-Resources/Patient-Registry/).

During the last few years, we focused our attention on this topic, proposing new targets, such as the glutamate racemase (Israyilova et al., 2016), new strategies, such as the inhibition of quorum sensing (Scoffone et al., 2016; Buroni et al., 2018) and new drugs (Scoffone et al., 2014), to fight *B. cenocepacia* infections. We found that the benzothiadiazole derivative C109 is highly effective against *B. cenocepacia* (Scoffone et al., 2015) and other Gram-negative and-positive bacteria, including *Mycobacterium abscessus* (Hogan et al., 2018). We were also able to identify the cellular target of C109 as the highly conserved cell division protein FtsZ (Hogan et al., 2018). This protein has recently emerged as a new promising target for the development of pharmacological agents against CF pathogens (Buroni et al., 2020). This not only justifies the broad-spectrum activity of C109, but also validates it as a robust molecule that hits an essential pathway, which is evolutionarily distant from its eukaryotic counterpart.

Due to the poor solubility of C109, we recently described the development of PEGylated nanocrystals in which the compound was stabilized with D-α-tocopheryl polyethylene glycol 1000 succinate embedded in hydroxypropyl-β-cyclodextrin (Costabile et al., 2020). This powder formulation allows its re-dispersion in water for *in vitro* aerosolization. The ability of these C109 nanocrystals to diffuse through artificial mucus and possess low toxicity toward human bronchial epithelial cells was also demonstrated (Costabile et al., 2020). The great potentiality of this formulation has been shown also by its activity against both planktonic and sessile *B. cenocepacia* strains, and by its efficacy in combination with piperacillin (Costabile et al., 2020).

Despite its promising activity and low toxicity, we previously showed that C109 can be extruded out of the cell by an RND efflux pump (Scoffone et al., 2015). For this reason, in the present work we synthesized and characterized more than 50 C109 derivatives, by performing a deep structure-activity relationship (SAR) analysis to screen for compounds less prone to efflux. The Minimal Inhibitory Concentration (MIC) of all the compounds and their activity against the purified FtsZ protein were assessed. The C109 resistance mechanism, chemical, metabolic and cellular stability were also studied at the single-cell level.

## Materials and Methods

### Chemical Synthesis of C109 Derivatives

The chemical synthesis of C109 derivatives is described in Supplementary Data.

### Bacterial Strains and Growth Conditions

*Burkholderia cenocepacia* strains, *Escherichia coli* ATCC 25922, *Pseudomonas aeruginosa* PAO1, and *Staphylococcus aureus* ATCC 25923 were grown in Luria-Bertani (LB) medium (Difco), if not differently specified, with shaking at 200 rpm, or on LB agar, at 37°C.

### MIC Determination and Checkerboard Assays

The effectiveness of C109 compound and of its derivatives against *B. cenocepacia* J2315, FCF19, and FCF22, *E. coli* ATCC 25922, *P. aeruginosa* PAO1, and *S. aureus* ATCC 25923 was assessed determining MICs in LB medium by the 2-fold microdilution method in U-bottom 96-well microtiter plates, and inoculating about 10^5^ CFU. The microtiter plates were incubated at 37°C for 48 (for *B. cenocepacia*) or 24 h (for all the other strains) and growth was determined by the resazurin method (Martin et al., 2006). A solution of resazurin sodium salt (Sigma Aldrich) was prepared at 0.01% in distilled water and filter-sterilized. Thirty microliter of resazurin solution were added to each well after 1 or 2 days of incubation at 37°C, and the microtiters were re-incubated at the same temperature for 4 h. The MIC was defined as the lowest concentration of the drug that prevented a change in color from blue to pink, which indicates bacterial growth.

Checkerboard assays of C109 or its derivatives with concentrations ranging from 0 to 128 μg/ml in combination with the efflux pump inhibitor PaβN MC-207.110 (0–128 μg/ml) were set up, as previously described (Saiman et al., 1996) and the results determined using the resazurin MIC method.

Three independent biological replicates were used for each MIC determination.

### Quantitative Reverse Transcription PCR (qRT-PCR) of RND-9 Efflux Pump and Nitroreductase Genes

Total RNA was extracted from *B. cenocepacia* J2315, FCF19, and FCF22 clinical isolates (1 × 10^9^ CFU) using the RiboPure Bacteria Kit (Ambion), following the manufacturer's instructions. A 30 min incubation of each sample with DNaseI (Ambion) was performed, following the manufacturer's protocol. One-microgram of total RNA was used for cDNA generation using the QuantiTect reverse Transcription kit (Qiagen) according to the manufacturer's instructions, but diluting the cDNA 1:2 before qRT-PCR.

qRT-PCRs were performed on *BCAM1946* gene using the primers BCAM1946Rtfor (5′- TGCTCGTCGTGATCCTGTTT-3′) and BCAM1946Rtrev (5′- CGAACAGCGTGAGCGTATTG-3′) and on *BCAL0539* using primers NitroIntFor (5′-GTGTCGCCGTATATCGA-3′) and NitroIntRev (5′-TTCTCGTCGCGGCCGAT-3′). All reactions were performed on a Rotor-Gene-6000 cycler (Corbett), using the Quanti-Tect SYBR Green PCR Kit (Qiagen), according to manufacturer's instructions. Cycling conditions were: 95°C for 15 min (1 cycle), 94°C for 15 s followed by 54°C for 30 s (for *BCAM1946*) or 40°C for 30 s (for *BCAL0539*) and 72°C for 30 s (40 cycles). A melting curve analysis was included at the end of each run. Each sample was spotted in triplicate and control samples without cDNA were included in each experiment. *BCAM0918* (*rpoD*) gene was used as reference gene with the primers 0918F (5′-GCCAACCTGCGTCTCGT-3′) and 0918R (5′-AACTTGTCCACCGCCTT-3′), using an annealing temperature of 50°C. The fold difference in gene expression between the FCF19 and FCF22 and J2315 strains was assessed using the comparative Ct-method (Livak and Schmittgen, 2001). The results are the average of three independent replicates. Mann–Whitney test was used to determine if differences in expression were significant (*P* < 0.05).

### FtsZ Proteins Expression and Purification and Enzymatic Assay

The gene *ftsZ* (*BCAL3457*) was amplified from *B. cenocepacia* FCF19 using the primers pet28presFtsZfor (5′-ATGGGTCGCGGATCCCTGGAAGTTCTGTTCCAGGGGCCCATGGAATTCGAAATGCTGGA-3′) and pet28ftsZrev (5′-TGCGGCCGCAAGCTTTCAGTCAGCCTGCTTGCGCA-3′). The gene *ftsZ* of *P. aeruginosa* was amplified from the genomic DNA using the primers ftsZPAOfor28a (5′-ATGGGTCGCGGATCCCTGGAAGTTCTGTTCCAGGGGCCCTTTGAACTGGTCGATAACAT 3′) and ftsZPAOrev28a (5′-TGCGGCCGCAAGCTTTCACAGGCCGGTGGCGACTAC-3′). The PCR products were cloned into the pET-28a vector (Novagen) using the In-Fusion HD Cloning kit (TaKaRa), according to the manufacturer's instructions.

All the recombinant FtsZ proteins were expressed at 20°C overnight in *E. coli* BL21(DE3) cells, upon induction with 0.5 mM IPTG, and purified using the same protocol assessed for the wild-type *B. cenocepacia* FtsZ (Hogan et al., 2018).

GTPase enzyme activity was determined using a pyruvate kinase/L-lactic dehydrogenase (PK/LDH) spectrophotometric coupled assay (Ingerman and Nunnari, 2005). The assays were performed at 30°C, in a reaction mixture containing 50 μM Hepes pH 7.5, 5 mM MgCl_2_, 5 mM KCl, 10 U PK/LDH, 0.25 mM NADH, 0.25 mM PEP, and 5–10 μM FtsZ, and initiated by the addition of 0.5 mM GTP. Alternatively, GTPase activity was determined by measuring the release of phosphate using the malachite green assay (Baykov et al., 1988).

Steady-state kinetic analysis was performed by assaying the activity at different GTP concentrations and parameters determined by fitting the data to the Michaelis-Menten equation using Origin 8 software. All experiments were performed in triplicate.

Initial Inhibition assays were performed at 100 μM of each compound (stock solution 20 mM in DMSO) and, for compounds significantly active, the inhibitory concentration (IC_50_) was determined. To this purpose, GTPase activity of FtsZ was evaluated in the presence of different concentrations of compounds, ranging from 0.5 μM to 100 μM, and IC_50_ value was determined by the Equation 1 using Origin 8 software:

(1) $$\begin{array}{l} A_{\left[I\right]}=A_{\left[0\right]}\times \left(1-\frac{\left[I\right]}{\left[I\right]+IC_{50}}\right) \end{array}$$

where A_[I]_ is the enzyme activity at inhibitor concentration [I] and A_[0]_ is the enzyme activity without inhibitor.

### Metabolic Transformation of C109 in *B. cenocepacia* Cultures

To identify the products of C109 transformation in the cellular environment, 200 ml *B. cenocepacia* J2315 culture were grown overnight at 37°C in LB medium, then 20 mg C109 were added. After 1.5 h, a chloroform extraction (100 ml × 3) was performed. Organic phase obtained from the extraction was evaporated, residues were resuspended in hexane/ethyl acetate 9:1 and subjected to flash column chromatography (Merck SiO2 60, 230–400 mesh). Visualization of metabolites was achieved under UV light at a wavelength of 254 nm.

The isolated metabolites were analyzed in UPLC/MS. The chromatographic analysis was performed with a JASCO X-LC (Lecco, Italy) system, coupled with a Thermo Fisher Scientific (Milan, Italy) LTQ XL HESI-MS/MS system. Chromatography was performed on a Waters Acquity column, 3 um particle size, 0.3 ml/min, gradient 10 min from 90:10 H_2_O/MeCN to 100% MeCN, then 4 min in 100% MeCN. The analyses were performed in full-scan from 150 and 2,000 u.m.a. in positive mode, and base peaks were analyzed with dependent scan method with @CID = 32 eV. Run were also monitored by recording the absorbance at 220 nm.

### Nitroreductase BCAL0539 Expression, Purification, and Characterization

In order to obtain the *B. cenocepacia* putative nitroreductase (BcNR), the *BCAL0539* gene was amplified using the primers pet28BC0539for (5′-ATGGGTCGCGGATC*CCTGGAAGTTCTGTTC*CAGGGGCCC-3′) and pet28BC0539rev (5′-TGCGGCCGCAAGCTTTCAAGCGAAAAAGCGCGCG-3′), and PCR products cloned into the pET-28a vector by the “In-Fusion® HD Cloning.” To facilitate the purification process, the forward primer was designed to insert the sequence encoding the cleavage site of the PreScission Protease (GE Healthcare) downstream the N-terminal His-tag sequence (italicized in the primer sequence). Best protein expression was achieved in *E. coli BL21*(DE3) cells, by induction with 0.5 mM IPTG overnight at 25°C. Cells were frozen at −20°C until use.

Frozen cells were resuspended in 50 mM potassium phosphate pH 8.0, 500 mM KCl (buffer A) containing 1 mM phenylmethylsulfonylfluoride, lysed by sonication, and clarified by centrifugation at 50,000 × *g* for 1 h. Cell free extract was then applied on a HisTrapFF Crude (1 ml, GE Healthcare) column, washed with 50 mM imidazole in buffer A, then BcNR was eluted with 250 mM imidazole in the same buffer. Proteins were dialyzed against 50 mM potassium phosphate pH 8.0, 150 mM KCl (buffer B), containing 1 mM DTT, treated with PreScission Protease to remove the N-terminal histidine tag, then further purified by size exclusion chromatography on a HiLoad 16/60 Superdex-75 column (GE Healthcare) equilibrated in buffer B. Protein was concentrated to 10 mg/ml and stored at −80°C until use. As BcNR was produced as a flavoprotein, we explored whether the prostetic group is covalently bound to the enzyme. To this purpose, 0.5 μl of the protein (2 mg/ml) were incubated at 100°C for 10 min, and centrifuged for 20 min at 12,000 × *g*. The denaturated protein was resuspended in the same initial volume of 2% SDS, and the spectra of both supernatant and resuspended pellet were recorded.

Enzyme activity assay was determined at 37°C using 4-nitrobenzoic acid or C109 as a substrate and NADPH as cofactor, by measuring the decrease in absorbance at 340 nm of NADPH (ε = 6,220 M^−1^ cm^−1^). The reaction mixture typically contained 50 mM potassium phosphate pH 8.0, 50 mM KCl, 5–10 μM BcNR, and reactions were started by adding the substrate. Steady-state kinetics parameters were determined by assaying the enzyme at variable concentrations of substrate (10–500 μM). The experiments were performed in triplicate, and the kinetic constants *K*_m_ and *k*_cat_ determined by fitting the data to the Michaelis-Menten equation using Origin 8 software.

To determine the BcNR metabolite of C109, 5 mg of compound were incubated with 10 mg of BcNR in 50 mM potassium phosphate pH 8.0, 200 μM NADPH, at 37°C, in a final volume of 35 ml for 1 h under agitation. The reaction mixture was then partitioned between water and dichloromethane (DCM), the aqueous layer was extracted with DCM, and combined organic layer washed with brine and dried over Na_2_SO_4_. Solvent was removed under reduced pressure and residue purified by flash column chromatography with hexane-ethyl acetate 8:2 as eluent. The isolated metabolites were analyzed in UPLC/MS, as described above. Analysis were performed in full-scan from 90 and 1,000 u.m.a. and base peaks were analyzed with dependent scan method with @CID = 28 eV.

### Time-Lapse Microscopy

*B. cenocepacia* J2315 was cultured over-night in Middlebrook 7H9 broth, supplemented with 10% (v/v) DS enrichment (20 g dextrose, 8.5 g sodium chloride in 1 L water) and 5% (v/v) of a casamino acids solution (10 g casamino acids in 100 ml water). Cells were diluted 10-fold in the same fresh 7H9 medium, and 5 μl of the diluted cell suspension were inoculated into a custom-made microfluidic *hexa*-device, as previously described (Manina et al., 2019). Silicone tubing (0.76 mm ID) was connected to each inlet and outlet ports of the *hexa*-device to enable medium circulation. Two 50 ml syringes were connected to the inlet tubing, and prewarmed 7H9 medium was pumped through the microfluidic device at 15 μl/min. The outlet tubing were inserted into a waste receptacle containing bleach. The syringes were switched to 7H9 medium containing C109 (100 μg/ml) when appropriate. Bacteria were imaged in phase contrast, using a UPLFLN100XO2/PH3/1.30 objective (Olympus) and a high-speed sCMOS camera, with the help of an automated epifluorescence inverted microscope (DeltaVision Elite, GE Healthcare). The microscope was equipped with an environmental chamber maintained at 37°C. Images were recorded at 20-min intervals and up to 46 h. Exposure conditions were as follows: phase 50% T, 150 ms. In each experiment, 190 XY fields were imaged. The same experimental set-up was carried out twice.

### Single-Cell Image Analysis

Manual segmentation of individual cells and analysis of image stacks were performed using the ImageJ 1.52a software. The Selection Brush Tools was used to draw polygons corresponding to the shape of individual cells and to extract the cell planar area (μm^2^). Measurements of the cell area were repeated throughout the generation time of individual cells, to calculate the growth rate by fitting an exponential curve. Division and lysis events were scored from the total number of cells constituting a microcolony before and during both the first and second exposure to C109, and used to calculate both division and lysis rates, as previously described (Manina et al., 2015).

## Results

### Chemistry and Structure-Activity Relationship of C109 Derivatives

During our research in the field of the discovery of new anti-tuberculosis and anti-leprosy agents, we synthesized broad spectrum ortho-nitrodithiocarbomoyl heterocycles and analogs (Makarov et al., 2006). As a continuation of this research, we investigated the activity of some of these compounds on *B. cenocepacia* and surprisingly only compound C109 showed significant activity in whole cell screening (Scoffone et al., 2015). So, the primary aim of our current study was to improve the chemical properties of C109 in order to find new derivatives with lower MIC values toward *B. cenocepacia* and/or other bacteria, lower toxicity and, possibly, not extruded out of the cell by efflux mechanisms. To study structure activity and to design new active derivatives of C109, we used known medicinal chemistry methods to synthesize a broad spectrum of compounds and to probe their activity.

After screening 51 C109 derivatives (Supplementary Table 1), 23 compounds that showed inhibitory activity were examined to understand structure activity relationship.

The primary compound, C109, is a methyl [(4-nitro-2,1,3-benzothiadiazol-5-yl)thio]acetate (Scoffone et al., 2015). As a first step, we investigated the activity of several heterocyclic compounds having methylthioacetate moiety in ortho position to nitro group and observed that 2,1,3-benzothiadiazol scaffold is essential to preserve antibacterial activity. Also, our efforts to change methylthioacetate moiety to other sulfur containing nucleophilic substituents, such as dithiocarbamates, rhodano, imidothiocarbamate did not lead to any compounds with significant activity. Therefore, all other modifications have been limited with efforts to modify the nitro group and methylthioacetate side chain. The nitro group was explored to understand the essentiality of this reactive moiety. All the compounds lacking this group failed to be active both against the FtsZ activity and all the bacteria tested, confirming its essentiality.

Another way to modify the C109 compound was achieved through the synthesis of derivatives close to the original molecule, by modifying the size or hydrophobicity of the side chain, and introducing different moieties or atoms. This part of the molecule was explored as it can influence not only the binding of the compound to the molecular target FtsZ, but also the permeability across cell wall, as well as the affinity for efflux systems. For instance, the corresponding ethyl acetate derivative (11426142) partially lost activity against both bacteria and the purified enzyme (Table 1). The same was observed in the case of exchange of one proton inside chain on methyl group (11826110). We also observed completely loss of activity in the case of exchange of sulfur atom to oxygen (11726041), or nitrogen (11726042), or side chain length extension (10226047) (Table 1).

**Table 1:** Chemical structure, MICs and IC_50_ of C109 and of selected derivatives.

**Molecule**	**Chemical structure**	**MIC (μg/ml)**				Inhibition of Bc FtsZ*	
		***B. cenocepacia* J2315**	***E. coli* 25922**	***S. aureus* 25923**	***P. aeruginosa* PAO1**	**At 100 μM**	**IC_**50**_ (μM)**
C109	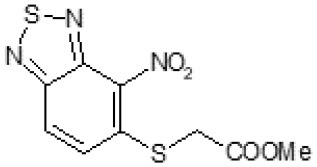	8	8	4	128	Yes	8
10026149	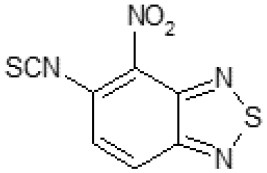	32	16	2	16	Yes	20
10126130	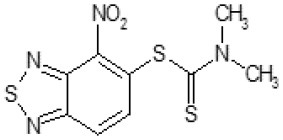	>128	512	32	>128	Yes	10
10226047	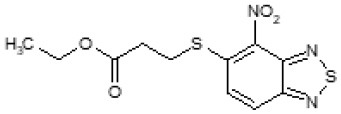	>512	>512	>512	>128	No	-
10626056	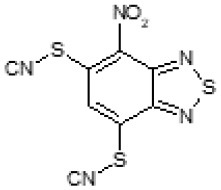	>64	>512	8	>128	Yes	52
10726015	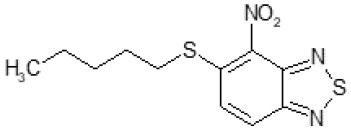	>64	>512	512	>128	No	-
11026176	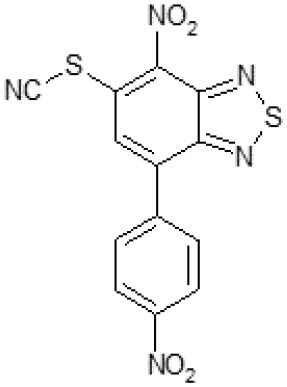	>512	>512	4	>128	Yes	17
11026177	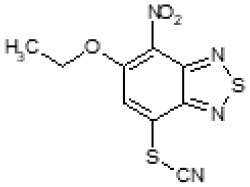	>512	>512	4	64	Yes	19
11126009	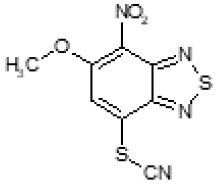	>128	>512	8	>128	Yes	4
11126010	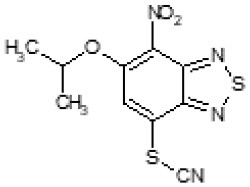	>128	>512	4	>128	Yes	15
11126015	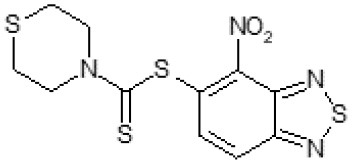	>128	>512	32	>128	Yes	36
11426142	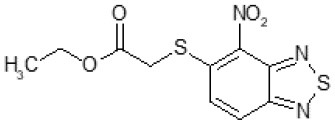	32	8	4	32	Yes	21
11426177	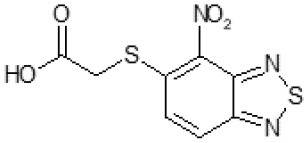	>128	>512	256	ND	No	–
11626109	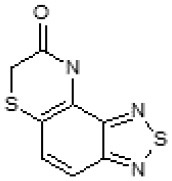	512	>512	>512	ND	No	–
11726041	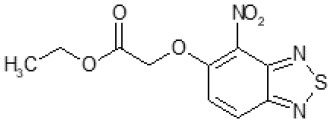	>256	>512	>256	ND	No	–
11726042	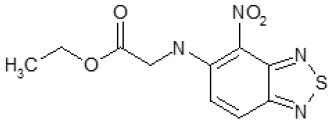	>256	>512	>256	ND	No	–
11726256	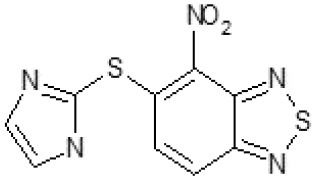	128	128	8	>256	No	–
11726257	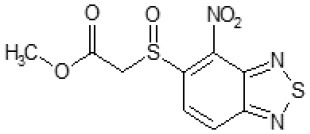	16	8	8	128	Yes	3
11726258	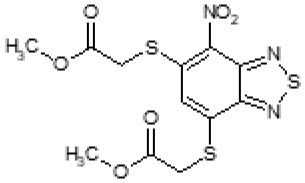	256	>256	256	>256	Yes	15
11826109	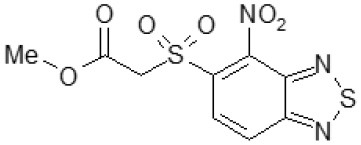	256	128	16	>256	Yes	30
11826110	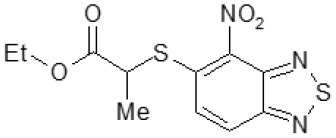	16	32	32	>256	No	–
11826363	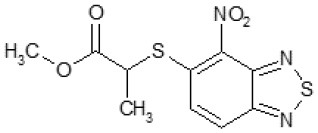	16	16	64	256	Yes	30
11926141	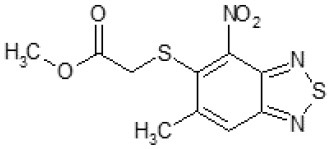	>256	>256	32	>256	Yes	100
11926142	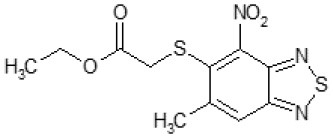	>256	>256	32	>256	Yes	100

ND, Values Not Detected because of the resistance detected in the other bacteria. * Burkholderia cenocepacia purified FtsZ protein.

Then, we explored typical metabolic processes such as hydrolysis, oxidation and reduction during which C109 can be transformed into its active metabolites. Hydrolysis followed by free acid formation led to the isolation of compound 11426177 (Table 1). Although this compound can be easily transferred to water soluble salt, unfortunately it is not active against *B. cenocepacia*. During oxidation process, we isolated two compounds with different level of sulfur oxidation. The result of mono oxidation, the sulfinyl derivative 11726257, showed an activity on *B. cenocepacia* similar to C109 (Table 1). A deeper oxidation of sulfur, which resulted in the sulfonyl derivative 11826109 showed loss of activity. We also studied the reduction process of nitro group of xenobiotics: several chemical methods of reduction were explored but, surprisingly, in all cases we isolated only products of the reduction followed by cyclisation of the thiozine ring with formation of the interesting tricyclic compound 7*H*-[1,2,5]thiadiazolo[3,4-*f* ][1,4]benzothiazin-8(9*H*)-one 11626109. This considerable modification of C109 structure caused loss of activity (Table 1).

Collectively these data show that the C109 molecule is very sensitive to any change, and that its structure is therefore most likely “terminal” for this class of compounds and for the activity against *B. cenocepacia*.

### C109 Derivatives Exhibit a Broad Spectrum Efficacy

Overall, none of the derivatives showed better MIC values against *B. cenocepacia* than C109 (Table 1 and Supplementary Table 1). To check whether the compounds were effective against other Gram-negatives, the MICs against *E. coli* were evaluated, as described in Material and Methods, showing results very similar to those achieved for *B. cenocepacia* (Table 1). The activity of the identified compounds was assessed also against *P. aeruginosa* and the Gram-positive *S. aureus*. Seven of them (10626056, 11026176, 11026177, 11126009, 11126010, 11126015, and 11726256) showed activity only against *S. aureus*, indicating that probably these molecules cannot enter the Gram-negative cells, due to the different composition of the cell wall and the presence of the outer membrane. However, compounds 10026149 and 11426142 had better MIC values than C109 against *P. aeruginosa* (16 and 32 μg/ml, respectively vs. 128 μg/ml).

To extend the basic antimicrobial inhibitory activity screening, we also determined the ability of all the C109 derivatives, showing activity against at least one of the microorganisms, to inhibit their cellular target, i.e., the cell division protein FtsZ. The IC_50_ of the selected compounds was assayed on the *B. cenocepacia* FtsZ recombinant protein, as previously described (Hogan et al., 2018). As expected, all the compounds showed inhibitory activity, having an IC_50_ ranging from 3 μM (11726257) to 100 μM (11926141 and 11926142, Table 1), thus confirming that the resistance identified is probably due to either limited cell penetration or increased efflux.

Being that 11426142 was effective against all the microorganisms tested, including *P. aeruginosa*, and showing an IC_50_ against FtsZ of 21 μM (Table 1), we also determined its toxicity against a CF bronchial epithelial (CFBE41o-) cell line using the MTT assay, as previously described (Hogan et al., 2018). The toxic concentration at 50% (TC_50_) of C109 on these cell lines is about 75 μM (Hogan et al., 2018; Costabile et al., 2020), while 11426142 showed a TC_50_ of 50 μM (data not shown), confirming once more that C109 remains the best compound in our hands.

### Analysis of C109 Resistant Strains

As we previously described that a mechanism of resistance to C109 relies on efflux mediated by the RND-9 transporter (Scoffone et al., 2015), we decided to investigate the resistance of *P. aeruginosa*, being the MIC of C109 equal to 128 μg/ml. A checkerboard assay, using increasing concentrations of C109 (from 0 to 128 μg/ml) in combination with the efflux pump inhibitor PaβN MC-207.110, was set up as described in Materials and Methods. The use of the efflux pump inhibitor PaβN MC-207.110 (Pagès et al., 2005) in combination with the C109 molecule showed a synergistic effect. In fact, in the presence of 16–128 μg/ml of MC-207.110, the C109 MIC decreased to 1 μg/ml (data not shown). These data clearly indicate that an efflux mechanism is responsible for the resistance of *P. aeruginosa* to C109.

**Figure 1:**
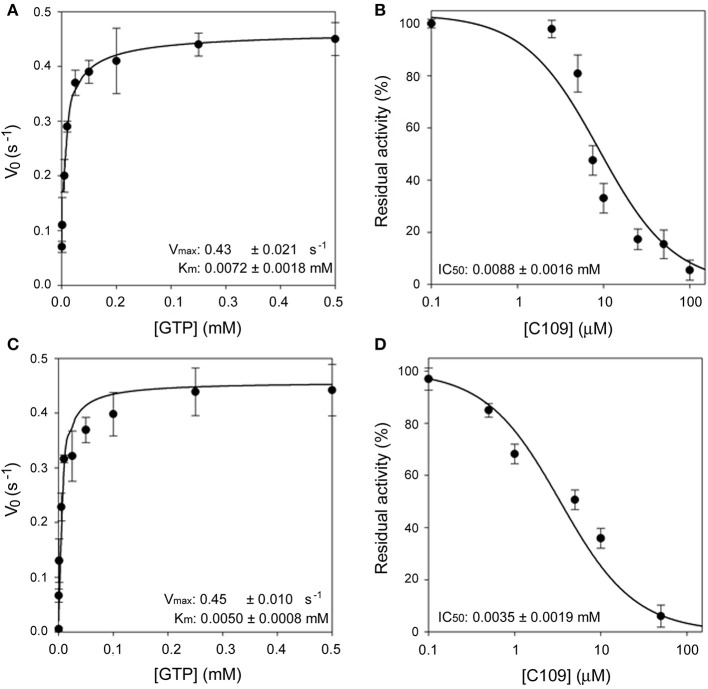
Enzymatic characterization of FCF19-FtsZ and PAO1-FtsZ. **(A)** Steady state kinetic analysis of FCF19-FtsZ mutant as a function of GTP. **(B)** IC_50_ determination of C109 toward FCF19-FtsZ mutant. **(C)** Steady state kinetic analysis of PAO1-FtsZ mutant as a function of GTP. **(D)** IC_50_ determination of C109 toward PAO1-FtsZ.

Moreover, the *ftsZ* gene from *P. aeruginosa* PAO1 was cloned and expressed in *E. coli* using the same protocol followed for FtsZ from *B. cenocepacia* (see Materials and Methods). The purified PAO1-FtsZ showed kinetic parameters comparable to the ones of *B. cenocepacia* enzyme and the protein was sensitive to the effect of C109, being the IC_50_ even lower than toward *B. cenocepacia* FtsZ (3.5 ± 1.9 μM vs. 8.2 ± 1.3 μM), showing that the compound is able to inhibit the activity of this protein (Figure 1). As the two proteins share a 53% amino acid sequence identity, this result was not unexpected but further confirmed that the conserved FtsZ protein is the cellular target of C109.

Next, we decided to investigate the origin of resistance to C109 in two clinical isolates of *B. cenocepacia*, namely FCF19 and FCF22 (Papaleo et al., 2010) for which C109 MIC is 128 and 256 μg/ml, respectively. Initially, we checked whether the gene encoding the C109 target, *ftsZ*, was mutated in these clinical isolates. Indeed, three mutations were found in the *ftsZ* gene of FCF19 strain, which lead to the following amino acidic changes: Pro347Gln; Ala348Pro; Gln349His. In contrast, no mutations were found in the *ftsZ* gene of FCF22 strain compared to the sequence of the reference strain J2315. Consequently, FCF19-FtsZ was expressed and purified in the same conditions of the wild-type FtsZ and showed to have kinetic parameters identical to those of the wild-type FtsZ (Figure 1). Moreover, the IC_50_ of C109 on FCF19-FtsZ resulted to be almost identical to the one for the wild-type protein (8.8 ± 1.6 μM), thus demonstrating that the mutated residues in FCF19-FtsZ are not responsible for the resistance phenotype.

Based on these findings, we sought whether the RND-9 efflux pump was implicated in the resistance phenotype of the two clinical isolates. We performed qRT-PCR analyses to check the level of expression of the RND-9 encoding gene. We found that the *BCAM1946* gene, encoding the RND-9 transporter, was overexpressed in both FCF19 and FCF22 clinical isolates of 12 and 20 times, respectively, compared to the wild-type J2315 strain (Table 2). As we previously isolated C109 resistant strains that carried mutations in the RND-9 regulator encoding gene (*BCAM1948*) or in the intergenic region, in between the first gene encoding RND-9 (*BCAM1947*) and the regulator (Scoffone et al., 2015), we sequenced the same region also in the two clinical isolates FCF19 and FCF22. However, no mutations were found in either the *BCAM1948* coding sequence or the intergenic region.

**Table 2:** MICs and RND-9 qRT-PCR results of *B. cenocepacia* FCF19 and FCF22 strains.

**Strain**	**C109 MIC (μg/ml)**	**C109 MIC in the presence of MC-207.110 (μg/ml)**	**10026149 MIC (μg/ml)**	**11426142 MIC (μg/ml)**	**RND-9 overexpression ± S.D**.
FCF19	128	8	64	32	12 ± 1.2
FCF22	256	16	256	32	20 ± 3.5

In order to determine whether the inhibition of the activity of efflux could affect the MIC of C109 in these strains, a checkerboard assay was set up using the compound C109 and the efflux pump inhibitor PaβN MC-207.110, as described above for *P. aeruginosa*. The MIC value of the C109 molecule in the presence of 128 μg/ml of the efflux inhibitor (Table 2) was reduced in both clinical isolates (from 128 μg/ml to 8 μg/ml for the strain FCF19 and from 256 to 16 μg/ml for FCF22), and comparable to the wild-type strain J2315. These data clearly indicate that an unidentified efflux mechanism is responsible for the resistance of these two clinical isolates.

Finally, the MIC of the C109 derivatives, having a lower MIC against *P. aeruginosa* (namely, 10026149 and 11426142), was determined also against the clinical isolates of *B. cenocepacia*, naturally resistant to C109, aiming to check whether they may be more effective and not subject to efflux. Both FCF19 and FCF22 clinical isolates showed resistance to the compound 10026149, whereas the MIC of the compound 11426142 was lower compared to the MIC of the compound C109 (32 μg/ml for both strains, Table 2). This value, which is identical to the MIC against *B. cenocepacia* J2315, did not change in the presence of different concentrations (0–128 μg/ml) of the MC-207.110 efflux inhibitor. In contrast, the MIC of 11426142 decreased from 32 to 2 μg/ml in the presence of MC-207.110 when the checkerboard assay was carried out using *P. aeruginosa*.

### Metabolic Transformation of C109 Upon Contact With *B. cenocepacia* Cells

**Figure 2:**
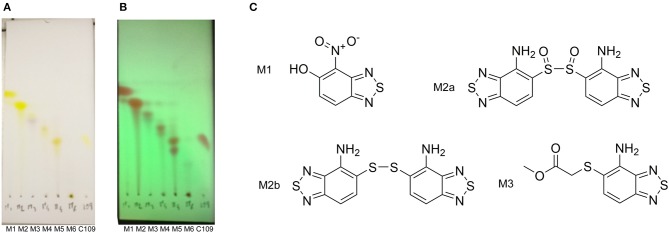
Identification of the putative C109 metabolites produced by *B. cenocepacia* cells. The unstained **(A)** and UV light visualized **(B)** TLC analysis of the chromatographic fractions of cell cultures extract highlighted the presence of different compounds. Four compounds were identified by UPLC-MS analysis (Supplementary Figures 1–5) as potential C109 metabolites **(C)**.

The TLC of the chromatographic fractions of C109 metabolites (Figure 2) showed that at least 6 different compounds could be isolated. All these fractions were subjected to UPLC-MS analysis to characterize the compounds, allowing us to identify four putative C109 metabolites, present in fractions 1 (M1), 2 (M2a and M2b) and 3 (M3) (Figure 2 and Supplementary Figures 1–4). Moreover, fraction M5 partly contained untransformed C109 (Supplementary Figure 5), whereas it was not possible to identify the compounds present in fractions 4 and 6 (data not shown).

The identified metabolites should derive from hydrolysis of C109 between the sulfur atom and the benzothiazole or the methylacetate moieties, and from reductive reactions particularly on the nitro group of the compound. To verify if these conversions lead to the inactivation of the compound, the MIC of these metabolites was determined using the resazurin method. None of the metabolites retained an antimicrobial activity, being all the MIC values >256 μg/ml (data not shown).

### *B. cenocepacia* Nitroreductase BCAL0539 Has the Potential to Inactivate C109

**Figure 3:**
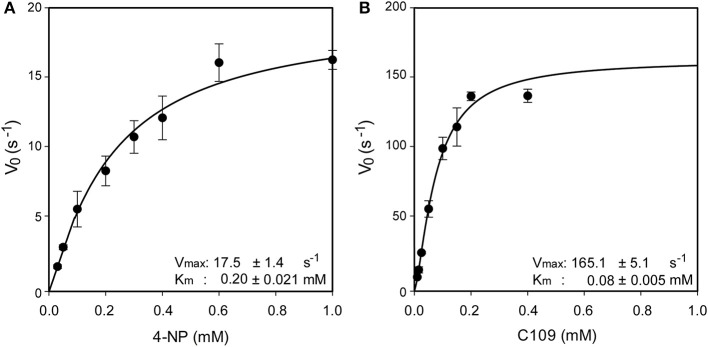
Steady state kinetic analysis of BcNR, using 4-nitrobenzoic acid **(A)** and C109 **(B)** as substrate.

Since in three out of four identified metabolites of C109 the nitro group resulted to be reduced to amino, leading to loss of activity, we investigated whether an enzyme could be responsible for such reaction. Searching in the *Burkholderia* Genome Database (www.burkholderia.com) we found the *BCAL0539* gene, which encodes a putative nitroreductase/4-nitrobenzoic reductase, belonging to the FMN—NAD(P)H-dependent nitroreductase family. To probe the role of this enzyme in the metabolism of C109, we produced the recombinant *B. cenocepacia* nitroreductase BCAL0539 (BcNR) protein in *E. coli*, and purified to homogeneity with a good yield (about 2 mg of protein per g of cells). The UV-vis spectrum of BcNR showed two peaks at 370 and 450 nm, demonstrating that the enzyme is expressed as a flavoprotein (Supplementary Figure 6). We found that the cofactor was not covalently linked to the enzyme because, after heat denaturation, the resuspended enzyme lost the two typical peaks of the flavin, which were instead present in the spectrum of the supernatant (Supplementary Figure 7). Since the enzyme is predicted to be a NAD(P)H dependent nitroreductase that uses 4-nitrobenzoic acid, we carried out a spectrophotometric activity assay, which follows the decrease in absorbance at 340 nm of the cofactor. Moreover, the enzyme activity was also assayed by using C109 as substrate. As shown in Supplementary Figure 8, BcNR is strictly NADPH-dependent, and is able to reduce both 4-nitrobenzoic acid and C109. Furthermore, the kinetic analysis showed that the enzyme is able to use C109 very efficiently, showing a specificity constant of 2063.7 ± 20.1 s^−1^ mM^−1^, about 20-fold higher than that for 4-nitrobenzoic acid, due to a 10-fold higher V_max_ and about a 2-fold lower K_m_ values (Figure 3 and Supplementary Table 2).

**Figure 4:**
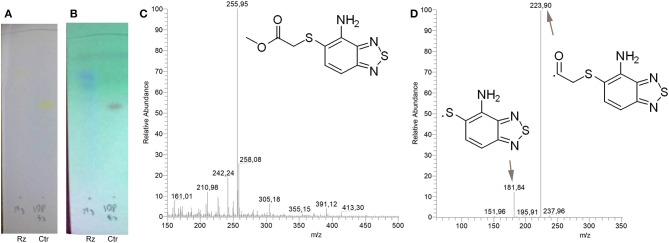
Identification of BcNR C109 metabolites. Unstained **(A)** and UV light visualized **(B)** TLC analyses of the BcNR reaction using C109 as substrate (Rz), compared with the control performed with the same reaction mixture but without NADPH, demonstrated that the enzyme is able to transform C109 compound. The MS analysis [**(C)** full mass, and **(D)** fragmentation pattern] of the purified reaction products demonstrated that BcNR catalyzes the reduction of the *nitro* group of C109.

To identify and characterize the C109 reaction product of BcNR, 5 mg of the compound were incubated for 1 h with the enzyme, in the presence of NADPH. The reaction mixture was then extracted with DCM, products isolated by silica gel chromatography and analyzed by mass spectrometry (Figure 4). Only a major metabolite was found, corresponding to the analogous amino metabolite of C109 M3, isolated in cell cultures (Figure 2), demonstrating that BcNR catalyzes the nitroreduction of the C109 compound.

To assess whether the resulting BcNR reduction product could inhibit *B. cenocepacia* growth, we carried out a resazurin-based assay. We set up three reactions: (i) a complete reaction in the presence of 1 mM C109; (ii) a reaction without the cofactor NADPH; (iii) a reaction without C109. All these reactions were assayed along with the C109 *nitro* derivative 11626109 (Table 1), revealing that the metabolite resulting from the complete reduction is not active against *B. cenocepacia* growth (as 11626109), while in the absence of NADPH the BcNR does not work, C109 is not reduced and, consequently, still active. All these data confirm a role of BcNR in the inactivation of the compound, thus suggesting a possible involvement of the enzyme in the resistance mechanism to C109.

For this reason, the *BCAL0539* gene expression was analyzed in the wild type J2315 and in the clinical isolates FCF19 and FCF22, which are resistant to C109 (see above). However, the *BCAL0539* gene was not expressed, either in the wild type or in the resistant strains, implying that the BcNR is not implicated in C109 inactivation and resistance mechanism.

### Time-Lapse Microscopy of *B. cenocepacia* Treated With C109 Reveals a Fraction of Possible Persistent Cells

The killing dynamics of *B. cenocepacia* with the compound C109 at the bulk-population level were shown to be concentration dependent and biphasic (Scoffone et al., 2015). Biphasic killing represents the typical pattern of drug persistence, whereby the initial linear mortality of the population is followed by a second phase where a subpopulation of cells is able to endure inhibitory drug concentrations in the absence of genetic mutations (Van den Bergh et al., 2017). In contrast to drug-resistant cells, which continue to replicate in the presence of the drug, drug-persistent cells either derive from pre-existing non-growing cells or slow down their growth during drug exposure, and their progeny is killed with kinetics similar to that of the initial population (Van den Bergh et al., 2017; Balaban et al., 2019). Possible mechanisms that can bring about heterogeneous responses to drugs, such as drug persistence, include uneven expression and partitioning of efflux systems among single bacterial cells (Pu et al., 2016; Bergmiller et al., 2017), Importantly, the mechanism of resistance to the C109 compound is mediated by the RND-9 efflux transporter (Scoffone et al., 2015). To examine the C109 killing dynamics of *B. cenocepacia* at the single-cell level, we carried out a time-lapse microscopy experiment, divided into several steps. Wild type *B. cenocepacia* J2315 cells were first seeded into a microfluidic device (Manina et al., 2019), and perfused with prewarmed 7H9 medium for 3 h, to allow the cells to adapt to the microfluidic environment. After a few events of cell division, the cells were exposed to 100 μg/ml of C109 (12.5X-MIC for the wild-type strain used in this work) for 4 h, equivalent to about 4 replication cycles. Cells were then perfused with C109-free 7H9 medium for 12 h, enabling the recovery and expansion of surviving cells. Finally, to understand whether the survivors had acquired genetic resistance, cells were exposed a second time to C109 (12.5X-MIC) for 24 h, equivalent to about 24 replication cycles. During the time-lapse imaging, cells were monitored every 20 min for a total of 46 h. The total number of cells, the single-cell area, and the number of division and lysis events were measured during each step of the experiment.

**Figure 5:**
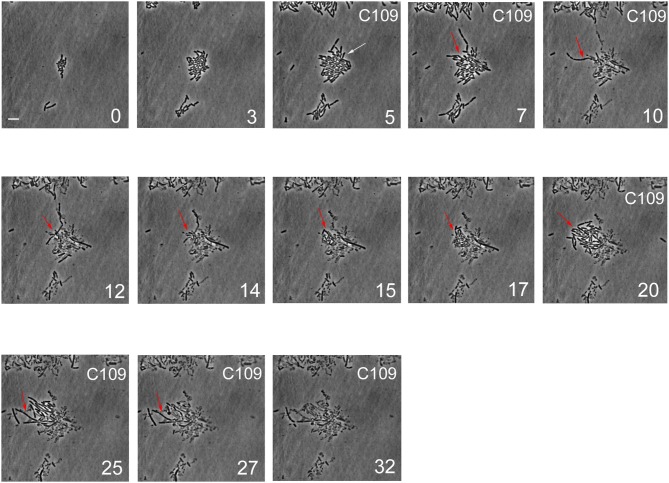
Single-cell imaging of *B. cenocepacia* cells treated with C109. Contrast-phase image stacks of *B. cenocepacia* J2315 during the time-lapse microscopy in the microfluidic chamber. The white arrow indicates a cell that after the C109 treatment died, while a filamentous cell is indicated by the red arrow. Time is indicated in h at the bottom-right corner of each picture. C109 treatment (12.5X-MIC) is indicated on the top-right corner. Scale bar in the first picture is 5 μm.

**Figure 6:**
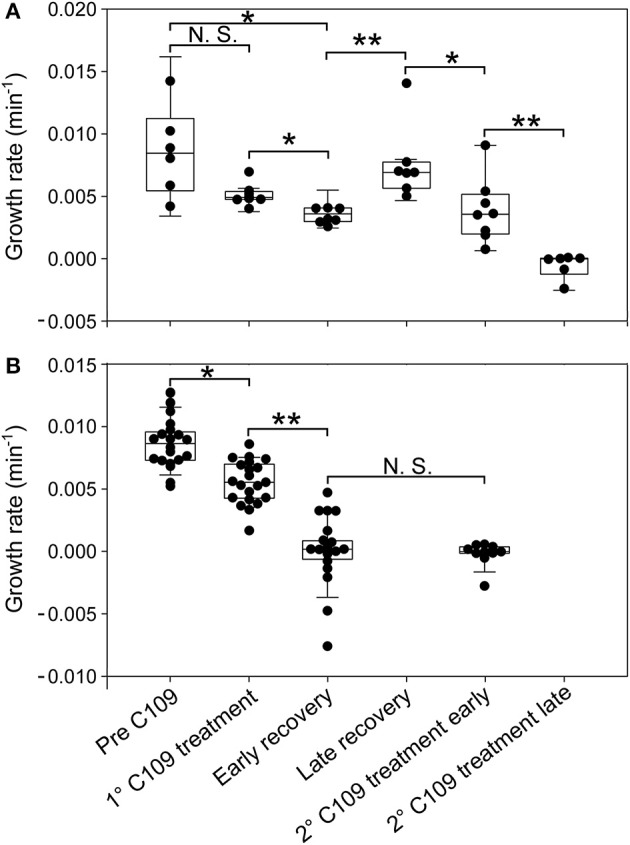
Single-cell analysis of growth rate for different cell categories. Single-cell growth rate of survivor cells **(A)** and cell dead by lysis **(B)** prior to and following C109 exposure (*n* = 30). Early recovery was defined between 6 and 10 h and late recovery was defined between 10 and 18 h, from the beginning of the experiment. 2nd C109 treatment early is defined between 19 and 22 h and 2nd C109 treatment late is defined between 22 and 46 h from the beginning of the experiment. The data shown are from 2 independent experiments. Black lines indicate mean ± SD. Asterisks denote significance by Paired samples *T*-test: N.S., not significant; *p ≤ 0.05 and **p ≤ 0.01.

**Figure 7:**
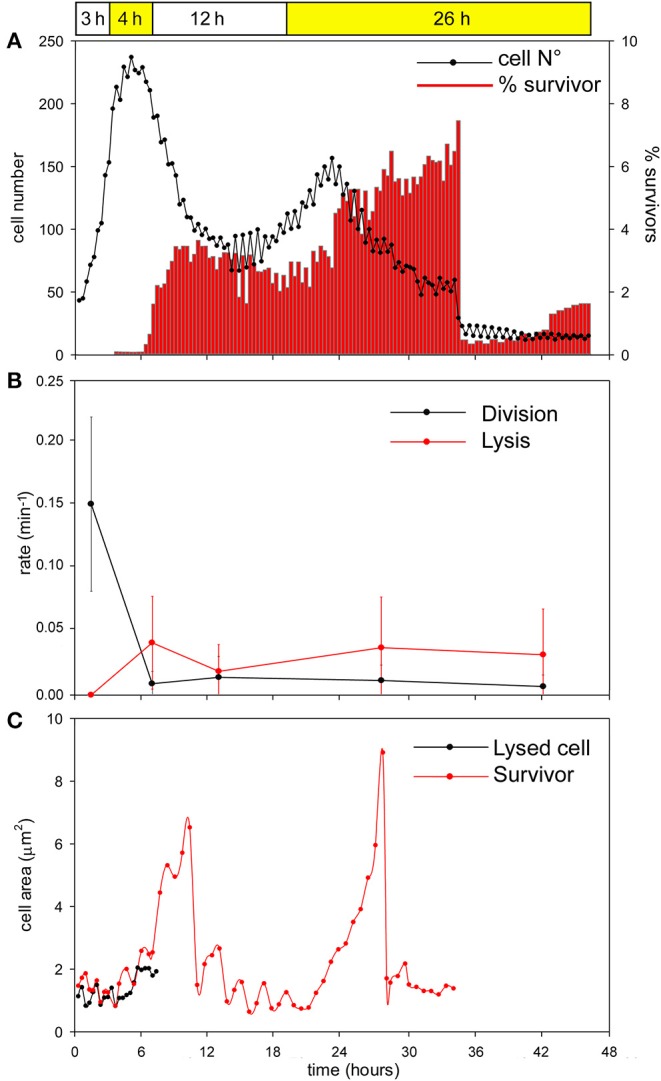
Single-cell analysis of *B. cenocepacia* cells treated with C109. **(A)** Total cell number (black line) and percentage of filamentous cells (red bars) for each time point during the time-lapse experiment. Data points are mean values of 8 different frames taken during two independent experiments. **(B)** Rates of cell division and lysis (mean ± SEM). Data points are mean values of different frames (*n* = 10) before, during and after C109 treatment, taken during two independent experiments. **(C)** Cell area measurements (μm^2^) of two-cells: cell dead by lysis (black line) and survivor filamentous cell (red line). The two cells are representative of 50 cells analyzed in two independent experiments.

During the first 3 h phase, *B. cenocepacia* cells were able to adapt to the microfluidic device and replicate every 60 min (Figures 5, 6). After the first treatment with C109 (12.5X-MIC), 70% of the cells died from lysis(Figures 5, 7A) and the growth rate and the number of cell divisions decreased (Figures 6, 7B), until cells stopped dividing (Figure 7B). During the recovery phase in fresh 7H9 medium, 3.5 % of the remaining intact cells (30%) were able to resume growth and the cell number and the division rate increased again (Figures 5, 7A,B). After, the cells that had survived and resumed growth were treated for the second time with C109 (12.5X-MIC), we found that the compound caused again a decline in cell population by 92%, and finally cells stopped dividing (Figure 7A). Importantly, during the second treatment with C109 the population derived from the subpopulation of survivors showed a lysis rate comparable to the lysis rate of the initial population subject to the first treatment with C109, implying the absence of stable genetic resistance in the survivors, and reminiscent of a phenotype conceivably ascribable to persistent cells (Figure 7B).

From the single-cell point of view, the *B. cenocepacia* cell population exhibited two different phenotypes in the presence of C109 compound. During the first treatment with C109, 70% of the cells slowly decreased their rate of growth and division (Figures 5, 6B, 7B), increasing their area of 2-fold, and then died by lysis (Figures 5, 7C). These results were expected and consistent with previous observations upon inhibition of an essential component of the cell divisome by treatment with C109, and in case of other FtsZ inhibitors (Scoffone et al., 2015; Hogan et al., 2018; Tripathy and Sahu, 2019). The lytic phenotype could be explained because FtsZ is an essential component of the cell divisome and controls the cell wall biosynthesis at the septum site (Yang et al., 2017). Interestingly, 3.5% of cells decreased their growth rates, but were still able to grow and underwent considerable filamentation, increasing the cell area up to 5-fold (Figures 5, 6A, 7C and Supplementary Movie 1). During C109 washout with the fresh medium, 30% of these filamentous cells started again to divide from both poles, decreasing the cell area to 1.5 μm^2^, which is comparable to the size of healthy wt cells (Figures 5, 6A, 7B,C and Supplementary Movie 1), while the other cells remained frozen to the initial state. To determine whether the cell population that expanded from the cells that survived the first treatment with C109 had become genetically resistant, we treated the cells a second time with the C109 compound for 24 h. The population showed again both phenotypes; namely, 94% of the cells stopped to divide during the treatment (Figures 5, 6A, 7A,B and Supplementary Movie 1), while a smaller population (about the 6% of the total cell number) showed again the elongated phenotype (Figures 5, 7C and Supplementary Movie 1). These cells were unable to divide but increased their area up to 10-fold (8–10 μm^2^) the normal size of the cell (Figures 5, 7C and Supplementary Movie 1). Then, these elongated cells stopped to increase their area and died during the exposure to C109 (Figures 5, 7C and Supplementary Movie 1).

These results indicate that *B. cenocepacia* cells exposed to C109 produce a subpopulation with a phenotype ascribable to possible persistent cells. In particular, these survivor cells remain elongated in presence of the compound but restore their normal size and life cycle once the C109 is removed, giving rise to a new non-filamentous population. The latter population, once treated again with C109, shows again both phenotypes, i.e., a fraction of cells (94%) that is again sensitive to C109 and a second fraction of elongated cells (6%) that is still sensitive to C109 extended exposure, and does not show any sign of resistance. Among a representative population of 3,000 elongated cells that formed during the first exposure to C109, 8 cells (0.27%) resumed normal growth during drug washout and, being sensitive to the second exposure to C109, were ascribable to possible persistent cells.

## Discussion

*Burkholderia spp* infections represent a problematic threat for CF patients. However, due to the low infection rate, the development of novel antimicrobials has been limited and remains an urgent priority for these patients (Regan and Bhatt, 2019). For this reason, in the past few years we identified and characterized the FtsZ inhibitor C109, which showed strong activity against *B. cenocepacia*, including several clinical isolates (Scoffone et al., 2015; Hogan et al., 2018). Moreover, this compound proved to be active against both planktonic and sessile *B. cenocepacia* strains, and against different other Gram-positive and -negative bacteria (Hogan et al., 2018; Costabile et al., 2020).

However, despite its great potential, this compound displays two main drawbacks. First, we showed that C109 is a substrate of the RND-9 efflux pump (Scoffone et al., 2015), and efflux pumps are known to be among the most common mechanisms of resistance to antibiotics in *B. cenocepacia* (Perrin et al., 2010). Second, the compound is characterized by the presence of a nitro group, which is considered a structural alert in a potential drug, due to its possible association with mutagenicity and genotoxicity (Nepali et al., 2019). In addition, this nitro group could be the substrate of modifying nitroreductase enzymes, which can alter the efficacy of the compound (Nepali et al., 2019).

In particular, we explored the effects of the typical metabolic reactions of hydrolysis, oxidation and reduction on the activity of C109. However, none of the synthesized derivatives showed improved potency. Similarly, the modification introduced to synthetize close derivatives, such as acetylation, methylation of the benzothiadiazole group, substitution of the sulfur atom with oxygen or nitrogen atom, or the extension of the side chain, gave no improvement.

It is noteworthy that the majority of the derivatives, showing worsened potency in *B. cenocepacia* and in *E. coli*, maintained a similar or improved activity in *S. aureus*, further confirming that the main drawback of these compounds is the difficulty to access the cell, or to be maintained into Gram-negative cells. Indeed, the main issue of these compounds is that they are good substrate of efflux pumps, as we demonstrated in both *B. cenocepacia* clinical isolates and *P. aeruginosa*.

The other drawback of our benzothiadiazole compound is the presence of an essential nitro group, that could be subjected to different modifications. It is noteworthy that modifications occurring to nitro group could lead to the inactivation of the drug (Nepali et al., 2019), whereas in the case of pro-drugs these modifications can cause their activation (Mori et al., 2017). Thus, to better characterize the behavior of C109, we investigated how it can be metabolized by *B. cenocepacia* cells. As expected, the main transformations taking place are reductive reactions on the nitro moiety, and hydrolytic reactions occurring between the sulfur atom linking the benzothiazole with the methylacetate. We were able to isolate and characterize certain C109 metabolites, but all of them were shown to be inactive, demonstrating that the compound is not subjected to any activation process. However, having found that C109 is intracellularly inactivated through nitroreduction, we looked for possible enzymes responsible for this reaction. We found that *B. cenocepacia* possesses a gene encoding the putative nitroreductase BcNR. However, we exclude that BcNR confers resistance to the compound, being its basal level of expression negligible. At this stage, we cannot exclude that there are other possible endogenous nitroreductases that could be responsible for the inactivation of C109 in *B. cenocepacia*, although none has been reported in the literature so far.

The C109 compound is also particularly susceptible to the action of the RND-9 efflux pump, which is over-expressed in two clinical isolates we tested (Papaleo et al., 2010; Scoffone et al., 2015). Remarkably, stochastic expression of efflux pumps can induce a fraction of the bacterial population to become persistent to drugs (Pu et al., 2016; Bergmiller et al., 2017). Furthermore, it has been recently proposed that recurrent drug-persistent infections, such as those caused by *B. cenocepacia* in CF patients, may promote the onset of genetic drug resistance (Balaban et al., 2019). Here we decided to assess the single-cell behavior of a clonal population of *B. cenocepacia* J2315 when confronted with the compound C109, by using quantitative time-lapse imaging. Although the majority of the bacterial population stopped dividing and died during treatment, consistent with the essentiality of FtsZ and with the mislocalization of FtsZ-GFP in *E. coli* cells treated with C109 (Hogan et al., 2018), a small fraction of *B. cenocepacia* cells was still viable and exhibited a considerable filamentous phenotype, which reflects the ability of a subpopulation to tolerate dysfunctional cytokinesis. During the recovery in C109-free medium, these surviving cells gave rise to a new population with normal cell size that, following a second treatment, was still sensitive to C109 and did not develop stable resistance to the compound. These results imply that these transient filamentous cells could be considered cells with a phenotype ascribable to possible persisters, which will be subjected to further investigations. Interestingly, we found that cells that survived the C109 compound originated from metabolically active cells, which had a growth rate comparable to drug-sensitive cells, as previously described in different bacterial species (Van den Bergh et al., 2017). Although non-growing populations are usually enriched with drug-persistent cells, the phenomenon of persistence is not exclusively linked to the absence of growth ahead of drug exposure, but is multifactorial and can also be associated with the environmental conditions; species-specific factors; pre-existing cell-to-cell phenotypic variation; the drug class and the dynamics of drug exposure (Van den Bergh et al., 2017; Balaban et al., 2019; Goormaghtigh and Van Melderen, 2019; Manina et al., 2019). Here we report that the single-cell growth rate prior to C109 exposure is not predictive of survival toward C109 compound in *B. cenocepacia*. At present, we cannot discriminate whether C109-survivors might result from the exposure to the compound, or might derive from a subpopulation having a distinct phenotype before exposure to the compound. In the future, it will be interesting to explore whether stochastic over-expression of RND-9 or of another efflux pump may be responsible for the onset of persistence in a subpopulation of *B. cenocepacia*. We think that a better understanding of this phenomenon may help to conceive a drug combination that makes C109 more effective against *B. cenocepacia*.

In conclusion, within this work, we thoroughly characterized the potential of the C109 scaffold for the development of new antimicrobial compounds against microbial species that are life-threatening for CF patients. Despite most of the derivatives being effective against the cellular target FtsZ *in vitro*, none showed improved activity against Gram negative bacteria. However, five of these derivatives (10026149, 11026176, 11026177, 11126010, 11426142) showed promising activity against the CF and nosocomial pathogen *S. aureus*, suggesting another possible application for this class of compounds.

## Data Availability Statement

All necessary datasets generated for this study are included in the article.

## Author Contributions

SB, VM, and GR contributed conception and design of the study. LC expressed and purified the proteins and performed enzymatic and metabolic assays. VS performed cloning, enzymatic assays, and time lapse experiments. GT expressed and purified the proteins and performed qRT-PCR. GB analyzed data. OR and NM synthesized and analyzed all tested compounds. AP performed MS assays and analysis. GM performed time lapse experiments. SB performed microbiological experiments. All authors contributed to manuscript revision, read, and approved the submitted version.

### Conflict of Interest

The authors declare that the research was conducted in the absence of any commercial or financial relationships that could be construed as a potential conflict of interest.
