# Supplementary material for: Chemical, Metabolic, and Cellular Characterization of a FtsZ Inhibitor Effective Against Burkholderia cenocepacia
Source: Front Microbiol. 2020 Apr 7;11:562. doi: 10.3389/fmicb.2020.00562 (PMC7154053; doi:10.3389/fmicb.2020.00562)
Supplement: Supplementary file 1 [file Data_Sheet_1.docx]

Supplementary Material

# Supplementary Data

**1.1 Chemical synthesis of C109 derivatives**

Scheme 1. Synthesis of 7-H-5-R-4-nitro-2,1,3-benzothiadiazole derivatives

*General Procedure for the synthesis of 7-H-5-R-4-nitro-2,1,3-benzothiadiazole derivatives*

**a)** At 20^o^C, solution of nucleophilic agents (30 mmol) and triethylamine (30 mmol) in 3 ml of ethanol was slow added by drop to a solution of 5-chloro-4-nitro-2,1,3-benzothiadiazole (27 mmol) in 25 ml of ethanol. The reaction mixture was heated at 50^o^C for 1 hour, cooled and dissolved by 75 ml of water. The solid was filtered off and washed by water.

*Methyl [(4-nitro-2,1,3-benzothiadiazol-5-yl)thio]acetate* (C109) Yield 63%. Mp. 120-3ºС (EtOH). Mass (EI), *m/z* (*I_relat_.*(%)): 286.3016 [M]^+^ (76). C_9_H_7_N_3_O_4_S_2_. ^1^H NMR (DMSO-d_6_): 3.63 (s, 3H, OCH_2_), 4.29 (s, 2H, CH_2_), 7.97 (d, 1H, *J* = 9.8 Hz, CH), 8.33 (d, 1Н, *J* = 9.8 Hz, CH) ppm.

*4-Nitro-2,1,3-benzothiadiazol-5-yl thiocyanate* (10026149) Yield 92%. Mp. 185-7ºС (EtOH). Mass (EI), *m/z* (*I_relat_.*(%)): 239.2485 [M]^+^ (63). C_7_H_2_N_4_O_2_S_2_. ^1^H NMR (DMSO-d_6_): 7.95 (d, 1H, *J* = 9.8 Hz, CH), 8.40 (d, 1Н, *J* = 9.8 Hz, CH) ppm.

*4-Nitro-2,1,3-benzothiadiazol-5-yl dimethyldithiocarbamate* (10126130) Yield 87%. Mp. 137-9ºС (EtOH). Mass (EI), *m/z* (*I_relat_.*(%)): 301.3836 [M]^+^ (57). C_9_H_8_N_4_O_2_S_3_. ^1^H NMR (DMSO-d_6_): 3.41 (s, 6H, N(CH_3_)_2_), 7.81 (d, 1H, *J* = 9.8 Hz, CH), 8.31 (d, 1Н, *J* = 9.8 Hz, CH) ppm.

*Ethyl 3-[(4-nitro-2,1,3-benzothiadiazol-5-yl)thio]propanoate* (10226047) Yield 56%. Mp. 89-91ºС (EtOH). Mass (EI), *m/z* (*I_relat_.*(%)): 314.3549 [M]^+^ (68). C_11_H_11_N_3_O_4_S_3_. ^1^H NMR (DMSO-d_6_): 1.27 (t, 3H, *J* = 5.2 Hz, CH_3_), 2.68 (t, 2H, *J* = 6.9 Hz, CH_2_), 3.25 (t, 2H, *J* = 6.9 Hz, CH_2_), 4.16 (q, 2H, *J* = 5.2 Hz, OCH_2_), 7.81 (d, 1H, *J* = 9.8 Hz, CH), 8.32 (d, 1Н, *J* = 9.8 Hz, CH) ppm.

*4-Nitro-5-(pentylthio)-2,1,3-benzothiadiazole* (10726015) Yield 34%. Mp. 116-8ºС (EtOAc). Mass (EI), *m/z* (*I_relat_.*(%)): 284.3719 [M]^+^ (93). C_11_H_13_N_3_O_2_S_2_. ^1^H NMR (DMSO-d_6_): 0.87 (t, 2H, *J* = 6.1 Hz, CH_2_), 1.36 (br m, 4H, CH_2_), 1.87 (m, 2H, CH_2_), 2.82 (t, 2H, *J* = 6.6 Hz, SCH_2_), 7.87 (d, 1H, *J* = 9.8 Hz, CH), 8.33 (d, 1Н, *J* = 9.8 Hz, CH) ppm.

*4-Nitro-2,1,3-benzothiadiazol-5-yl thiomorpholine-4-carbodithioate* (11126015) Yield 81%. Mp. 140-2ºС (EtOAc). Mass (EI), *m/z* (*I_relat_.*(%)): 359.4869 [M]^+^ (37). C_11_H_10_N_4_O_2_S_4_. ^1^H NMR (DMSO-d_6_): 2.66 (m, 4H, S(CH_2_)_2_), 3.54 (m, 4H, N(CH_2_)_2_), 7.73 (d, 1H, *J* = 9.8 Hz, CH), 8.29 (d, 1Н, *J* = 9.8 Hz, CH) ppm.

*Ethyl [(4-nitro-2,1,3-benzothiadiazol-5-yl)thio]acetate* (11426142) Yield 59%. Mp. 172-4ºС (EtOH). Mass (EI), *m/z* (*I_relat_.*(%)): 300.3283 [M]^+^ (45). C_10_H_9_N_3_O_4_S_2_. ^1^H NMR (DMSO-d_6_): 1.27 (t, 3H, *J* = 5.2 Hz, CH_3_), 3.91 (s, 2H, CH_2_), 4.18 (q, 2H, *J* = 5.2 Hz, OCH_2_), 7.80 (d, 1H, *J* = 9.8 Hz, CH), 8.33 (d, 1Н, *J* = 9.8 Hz, CH) ppm.

*Ethyl [(4-nitro-2,1,3-benzothiadiazol-5-yl)oxy]acetate* (11726041) Yield 44%. Mp. 131-3ºС (EtOAc). Mass (EI), *m/z* (*I_relat_.*(%)): 284.2617 [M]^+^ (39). C_10_H_9_N_3_O_5_S. ^1^H NMR (DMSO-d_6_): 1.21 (t, 3H, *J* = 5.2 Hz, CH_3_), 4.19 (q, 2H, *J* = 5.2 Hz, OCH_2_), 5.23 (s, 2H, CH_2_), 7.87 (d, 1H, *J* = 9.8 Hz, CH), 8.32 (d, 1Н, *J* = 9.8 Hz, CH) ppm.

*Ethyl N-(4-nitro-2,1,3-benzothiadiazol-5-yl)glycinate* (11726042) Yield 49%. Mp. 169-71ºС (EtOH). Mass (EI), *m/z* (*I_relat_.*(%)): 283.2770 [M]^+^ (51). C_10_H_10_N_4_O_4_S. ^1^H NMR (DMSO-d_6_): 1.31 (t, 3H, *J* = 5.2 Hz, CH_3_), 4.12 (s, 2H, CH_2_), 4.32 (q, 2H, *J* = 5.2 Hz, NCH_2_), 7.91 (d, 1H, *J* = 9.8 Hz, CH), 8.35 (d, 1Н, *J* = 9.8 Hz, CH) ppm.

*5-(1H-imidazol-2-ylthio)-4-nitro-2,1,3-benzothiadiazole* (11726256) Yield 76%. Mp. 213-5ºС (EtOH). Mass (EI), *m/z* (*I_relat_.*(%)): 280.3005 [M]^+^ (72). C_9_H_5_N_5_O_2_S_2_. ^1^H NMR (DMSO-d_6_): 7.67 (d, 1H, *J* = 9.8 Hz, CH), 7.79 (br s, 2H, CHCH), 8.32 (d, 1Н, *J* = 9.8 Hz, CH), 13.21 (br s, 1H, NH) ppm.

*Ethyl 2-[(4-nitro-2,1,3-benzothiadiazol-5-yl)thio]propanoate* (11826110) Yield 45%. Mp. 153-5ºС (EtOH). Mass (EI), *m/z* (*I_relat_.*(%)): 313.3549 [M]^+^ (78). C_11_H_11_N_3_O_4_S_2_. ^1^H NMR (DMSO-d_6_): 1.18 (t, 3H, *J* = 5.2 Hz, CH_3_), 1.44 (d, 3H, *J* = 7.6 Hz, CH_3_), 4.03 (q, H, *J* = 7.6 Hz, CH), 7.88 (d, 1H, *J* = 9.8 Hz, CH), 8.33 (d, 1Н, *J* = 9.8 Hz, CH) ppm.

*Methyl 2-[(4-nitro-2,1,3-benzothiadiazol-5-yl)thio]propanoate* **(**11826363**)** Yield 41%. Mp. 161-3 ºС (EtOH). Mass (EI), *m/z* (*I_relat_.*(%)): 299.3283 [M]^+^ (64). C_10_H_9_N_3_O_4_S_2_. ^1^H NMR (DMSO-d_6_): 1.44 (d, 3H, *J* = 7.6 Hz, CH_3_), 3.63 (s, 3H, OCH_3_), 4.00 (q, H, *J* = 7.6 Hz, CH), 4.08 (d, H, *J* = 7.6 Hz, CH), 7.88 (d, 1H, *J* = 9.8 Hz, CH), 8.33 (d, 1Н, *J* = 9.8 Hz, CH) ppm.

*Methyl [(6-methyl-4-nitro-2,1,3-benzothiadiazol-5-yl)thio]acetate* (11926141) Yield 32%. Mp. 172-4 ºС (MeOH). Mass (EI), *m/z* (*I_relat_.*(%)): 299.3283 [M]^+^ (71). C_10_H_9_N_3_O_4_S_2_. ^1^H NMR (DMSO-d_6_): 2.71 (s, 1H, CH), 3.63 (s, 3H, OCH_2_), 4.29 (s, 2H, CH_2_), 7.97 (d, 1H, *J* = 9.8 Hz, CH), 8.33 (d, 1Н, *J* = 9.8 Hz, CH) ppm.

*Ethyl [(6-methyl-4-nitro-2,1,3-benzothiadiazol-5-yl)thio]acetate* (11926142) Yield 67%. Mp. 181-3 ºС (EtOH). Mass (EI), *m/z* (*I_relat_.*(%)): 313.3549 [M]^+^ (59). C_11_H_11_N_3_O_4_S_2_. ^1^H NMR (DMSO-d_6_): 1.27 (t, 3H, *J* = 5.2 Hz, CH_3_), 2.68 (s, H, CH_3_), 3.91 (s, 2H, CH_2_), 4.18 (q, 2H, *J* = 5.2 Hz, OCH_2_), 7.85 (s, 1H, CH) ppm.

**b)** At 20^o^C, solution of 5-chloro-4-nitro-2,1,3-benzothiadiazole (20 mmol) in 25 ml acetonitryl was treated by mercaptoacetic acid (30 mmol) and potassium carbonate (30 mmol). After 3 hours the reaction mixture was dissolved by 100 ml of water and acidified by water solution of hydrochloric acid (36%) till pH ~ 2. The light yellow solid was filtered off and washed by water.

*[(4-Nitro-2,1,3-benzothiadiazol-5-yl)thio]acetic acid* (11426177) Yield 85%. Mp. 215-7ºС (EtOH). Mass (EI), *m/z* (*I_relat_.*(%)): 272.2751 [M]^+^ (44). C_8_H_5_N_3_O_4_S_2_. ^1^H NMR (DMSO-d_6_): 4.01 (s, 2H, CH_2_), 7.83 (d, 1H, *J* = 9.8 Hz, CH), 8.38 (d, 1Н, *J* = 9.8 Hz, CH) ppm.

**c**) Solution of methyl [(4-nitro-2,1,3-benzothiadiazol-5-yl)thio]acetate (C109) (20 mmol) in 20 ml of trifluoroacetic acid was cooled till 0 ^o^C and treated by 1 mL of 32% water solution of hydrogen peroxide. The reaction mixture was stand 30 min at 0 ^o^C, 3 hours at 20 ^o^C and was dissolved by 100 ml of water. The yellow solid was filtered off and washed by water.

*Methyl [(4-nitro-2,1,3-benzothiadiazol-5-yl)sulfinyl]acetate* (11726257) Yield 63%. Mp. 179-81ºС (EtOH). Mass (EI), *m/z* (*I_relat_.*(%)): 302.3011 [M]^+^ (69). C_9_H_7_N_3_O_5_S_2_. ^1^H NMR (DMSO-d_6_): 3.73 (s, 3H, OCH_3_), 4.07 (d, H, *J* = 13.2 Hz, CH), 4.55 (d, H, *J* = 13.2 Hz, CH), 8.41 (d, 1H, *J* = 9.8 Hz, CH), 8.80 (d, 1Н, *J* = 9.8 Hz, CH) ppm.

**d**) Solution of methyl [(4-nitro-2,1,3-benzothiadiazol-5-yl)thio]acetate (C109) (20 mmol) in 20 ml of trifluoroacetic acid was cooled till 0 ^o^C and treated by 1,5 mL of 32% water solution of hydrogen peroxide. The reaction mixture was stand 30 min at 0 ^o^C, 2 hours at 70 ^o^C, cooled and was dissolved by 100 ml of water. The yellow solid was filtered off and washed by water.

*Methyl [(4-nitro-2,1,3-benzothiadiazol-5-yl)sulfonyl]acetate* (11826109) Yield 63%. Mp. 123-5ºС (MeOH). Mass (EI), *m/z* (*I_relat_.*(%)): 318.3005 [M]^+^ (38). C_9_H_7_N_3_O_6_S_2_. ^1^H NMR (DMSO-d_6_): 3.64 (s, 3H, CH_3_), 4.57 (s, 2H, CH_2_), 7.97 (d, 1H, *J* = 9.8 Hz, CH), 8.41 (d, 1Н, *J* = 9.8 Hz, CH) ppm.

**e)** Solution of methyl [(4-nitro-2,1,3-benzothiadiazol-5-yl)thio]acetate (C109) (20 mmol) in 30 ml of ethanol was treated by tin(II) chloride dehydrate (150 mmol) at room temperature. The reaction mixture was stand for 24 hours and dissolved by 75 ml of water, and treated by 25% ammonia water solution til pH~11. This solution was extracted by chloroform (3 x 50 mL), organic fractions was dried by sodium sulfate and evaporated in vacuum. The residue was crystallized from ethylacetate.

*7H-[1,2,5]thiadiazolo[3,4-f][1,4]benzothiazin-8(9H)-one* (11626109) Yield 47%. Mp. 211-13ºС (MeOH). Mass (EI), *m/z* (*I_relat_.*(%)): 224.2769 [M]^+^ (76). C_8_H_5_N_3_OS_2_. ^1^H NMR (DMSO-d_6_): 3.42 (s, 2H, CH_2_), 7.47 (d, 1H, *J* = 9.8 Hz, CH), 7.74 (d, 1Н, *J* = 9.8 Hz, CH), 10.47 (s, 1H, NH) ppm.

Scheme 2. Synthesis of 5-R-7-R-4-nitro-2,1,3-benzothiadiazole derivatives

*General Procedure for the synthesis of 5-R-7-R-4-nitro-2,1,3-benzothiadiazole derivatives*

**a)** Solid 3,5-dichlorobenzene-1,2-diamine (20 mmol) was slow added by small portions to the solution thionyl chloride (140 mmol) and sulfuric acid (80 mmol). The reaction mixture was refluxed for 1 hour and cooled till 35 ^o^C, dissolved by 160 mmol sulfuric acid and treated by mixture of 65 mmol fumigating nitric acid and 60 mmol of sulfuric acid. The mixture was stand for 30 min at room temperature and poured into ice. Solid was filtered off, washed by water and crystallized from acetone.

*5,7-Dichloro-4-nitro-2,1,3-benzothiadiazole***.** Yield 78%. Mp. 153-5ºС. Mass (EI), *m/z* (*I_relat_.*(%)): 251.0626 [M]^+^ (96). C_6_HCl_2_N_3_O_2_S. ^1^H NMR (DMSO-d_6_): 7.57 (s, 1H,CH) ppm.

**b)** At 20^o^C, solution of 5,7-dichloro-4-nitro-2,1,3-benzothiadiazole (30 mmol) in 25 ml acetonitryl was treated by 60 mmol of potassium thiocyanate or by methyl mercaptoacetate (60 mmol) and potassium carbonate (70 mmol). After 3 hours the reaction mixture was dissolved by 100 ml of water, light yellow solid was filtered off and washed by water.

*7-Nitro-2,1,3-benzothiadiazole-4,6-diyl bis(thiocyanate)* (10626056)**.** Yield 49%. Mp. 167-9ºС. Mass (EI), *m/z* (*I_relat_.*(%)): 296.3240 [M]^+^ (43). C_8_HN_5_O_2_S_3_. ^1^H NMR (DMSO-d_6_): 8.35 (s, 1H, CH) ppm.

*Dimethyl 2,2'-[(7-nitro-2,1,3-benzothiadiazole-4,6-diyl)bis(thio)]diacetate* (11726258)**.** Yield 37%. Mp. 141-3ºС. Mass (EI), *m/z* (*I_relat_.*(%)): 390.4304 [M]^+^ (27). C_12_H_11_N_3_O_6_S_3_. ^1^H NMR (DMSO-d_6_): 3.69 (m, 6H, 3OCH_3_), 4.42 (m, 4H, 2CH_2_), 7.49 (s, 1H, CH) ppm.

**c)** At 0 ^o^C, solution of 5,7-dichloro-4-nitro-2,1,3-benzothiadiazole (30 mmol) in 25 ml corresponding alcohol was treated by 30 mml of freshly prepared sodium alkoxide. The reaction mixture was stand for 2 hours and dissolved by 75 ml of water. The precipitation was collected, washed by water and corresponding 5-alkoxy-7-chloro-4-nitro-2,1,3-benzothiadiazole used in the next step without additional purification.

**d)** Solid potassium thiocyanate (30 mmol) was added in one portion to the solution of 5-alkoxy-7-chloro-4-nitro-2,1,3-benzothiadiazole (25 mmol) in 25 ml ethanol at room temperature. After 3 hours the reaction mixture was dissolved by 100 ml of water, light yellow solid was filtered off and washed by water.

*6-Methoxy-7-nitro-2,1,3-benzothiadiazol-4-yl thiocyanate* (11126009)**.** Yield 85%. Mp. 143-5ºС. Mass (EI), *m/z* (*I_relat_.*(%)): 269.2745 [M]^+^ (63). C_8_H_4_N_4_O_3_S_2_. ^1^H NMR (DMSO-d_6_): 4.19 (s, 3H, OCH_3_), 8.21 (s, 1H, CH) ppm.

*6-Ethoxy-7-nitro-2,1,3-benzothiadiazol-4-yl thiocyanate* (11026177)**.** Yield 73%. Mp. 128-9ºС. Mass (EI), *m/z* (*I_relat_.*(%)): 283.3011 [M]^+^ (74). C_9_H_6_N_4_O_3_S_2_. ^1^H NMR (DMSO-d_6_): 1.42 (t, 3H, *J* = 5.1 Hz, CH_3_), 4.53 (q, 2H, *J* = 5.1 Hz, CH_2_), 8.18 (s, 1H, CH) ppm.

*6-i-Propoxy-7-nitro-2,1,3-benzothiadiazol-4-yl thiocyanate* (11126010)**.** Yield 47%. Mp. 116-8ºС. Mass (EI), *m/z* (*I_relat_.*(%)): 297.3277 [M]^+^ (70). C_10_H_8_N_4_O_3_S_2_. ^1^H NMR (DMSO-d_6_): 1.34 (d, 6H, *J* = 6.2 Hz, CH_3_), 5.12 (m, 1H, CH), 8.23 (s, 1H, CH) ppm.

# Supplementary Figures and Tables

## Supplementary Figures


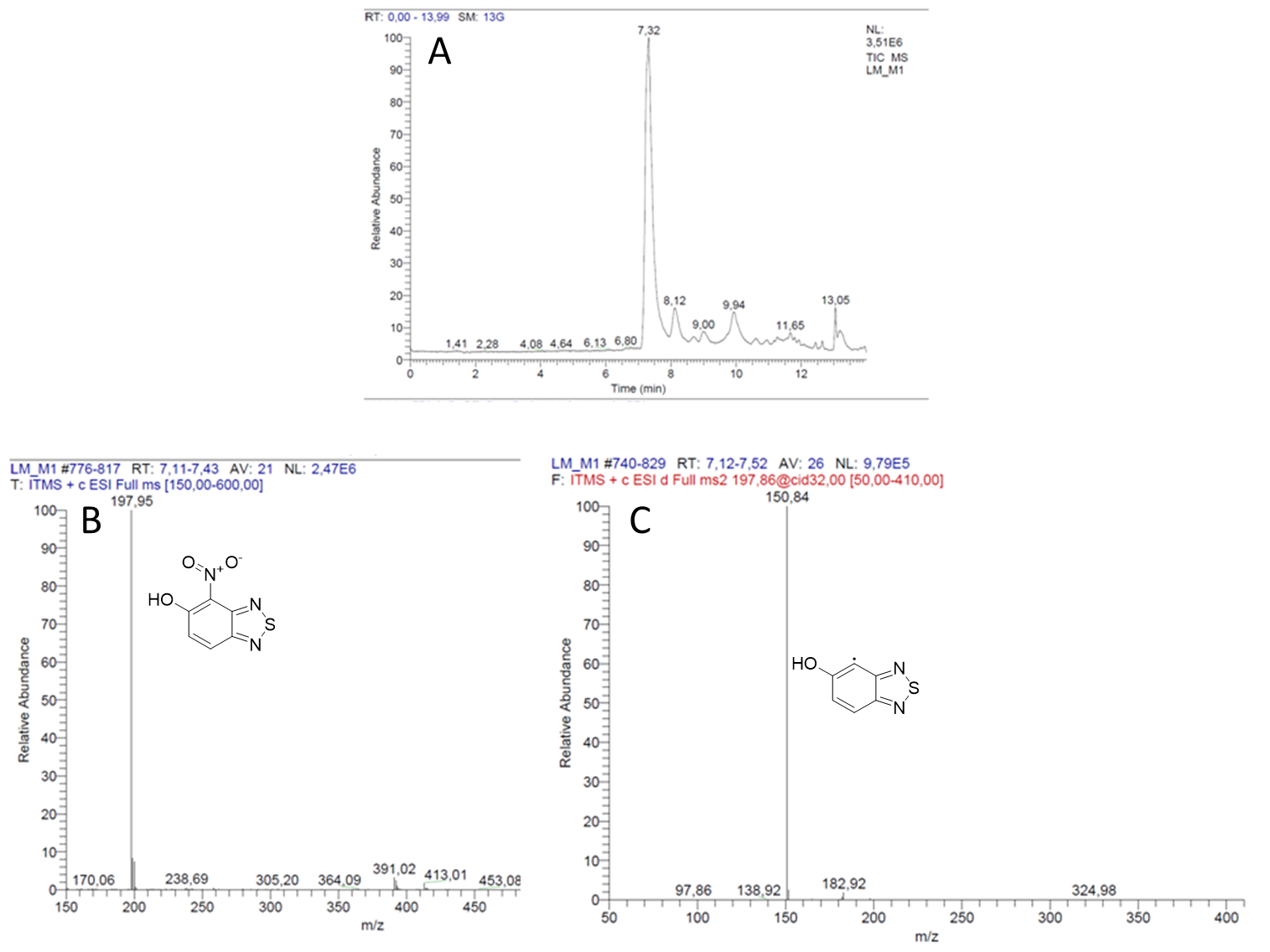


**Supplementary Figure 1.** UPLC-MS analysis of fraction M1 of the silica gel chromatography of the C109 treated *B. cenocepacia* culture extract. (A): chromatogram; (B) full-mass of the selected peak M1; (C) fragmentation pattern.


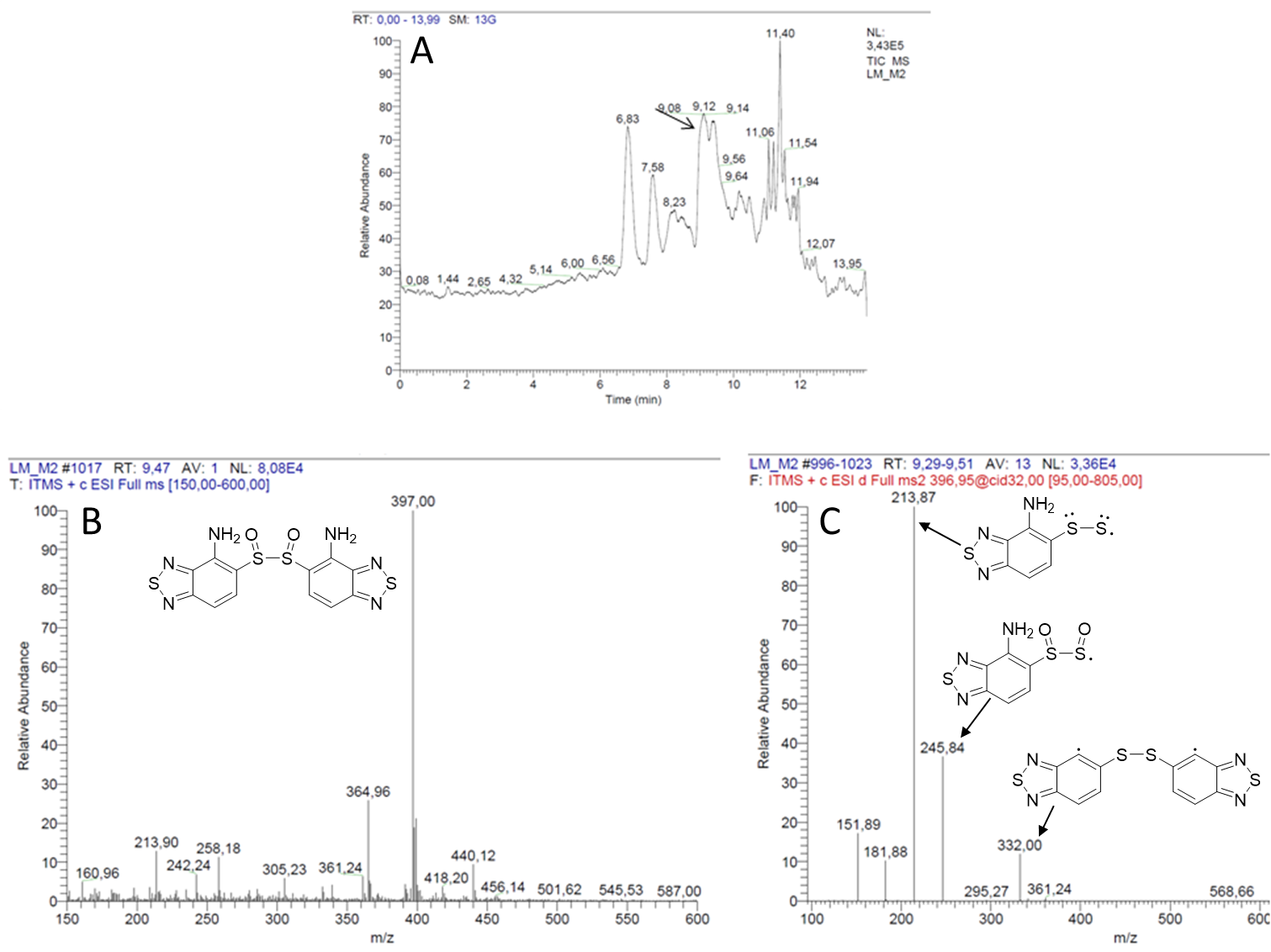


**Supplementary Figure 2.** UPLC-MS analysis of fraction M2 of the silica gel chromatography of the C109 treated *B. cenocepacia* culture extract. (A): chromatogram; (B) full-mass of the selected peak M2a; (C) fragmentation pattern.


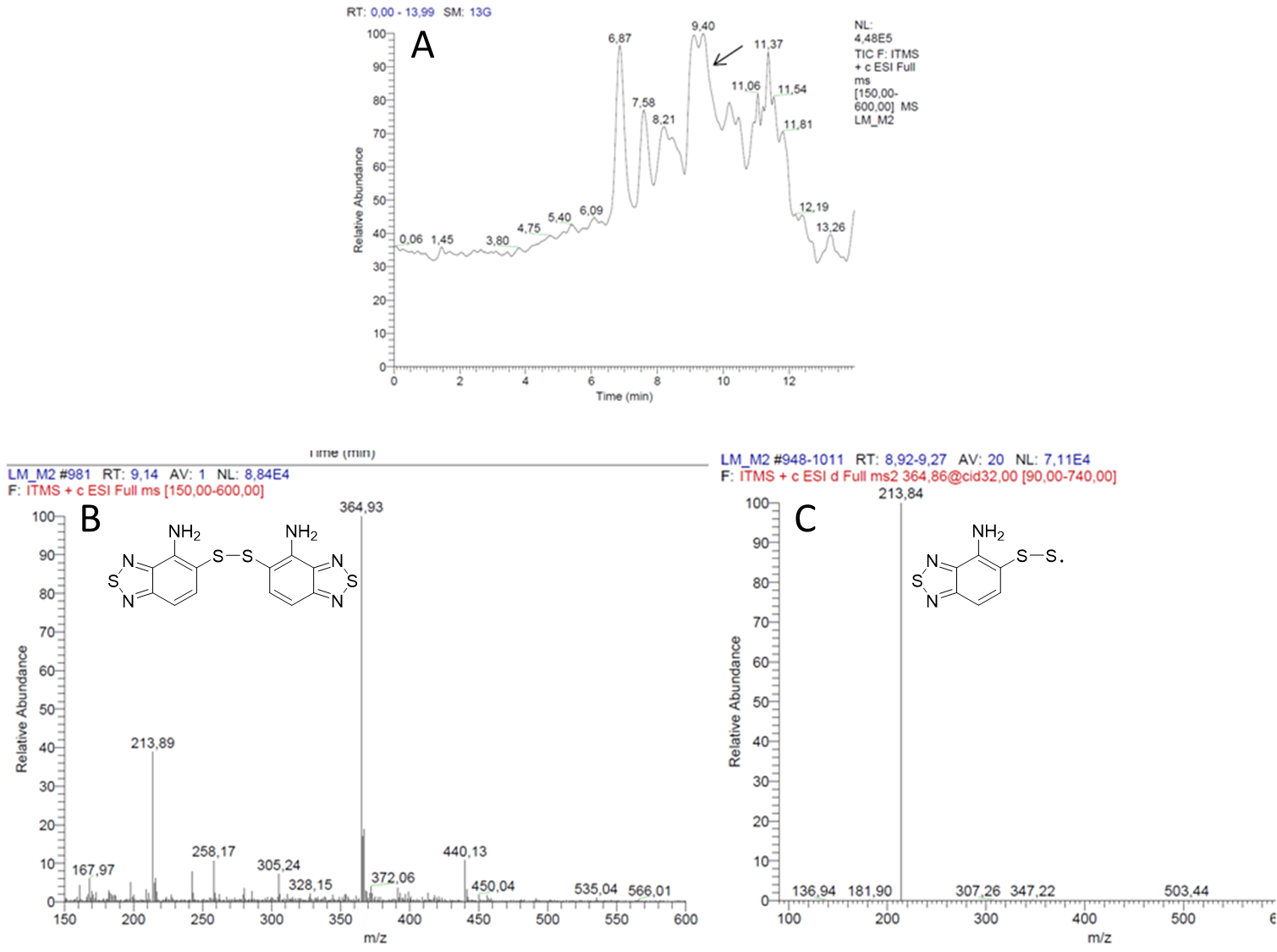


**Supplementary Figure 3.** UPLC-MS analysis of fraction M2 of the silica gel chromatography of the C109 treated *B. cenocepacia* culture extract. (A): chromatogram; (B) full-mass of the selected peak M2b; (C) fragmentation pattern.


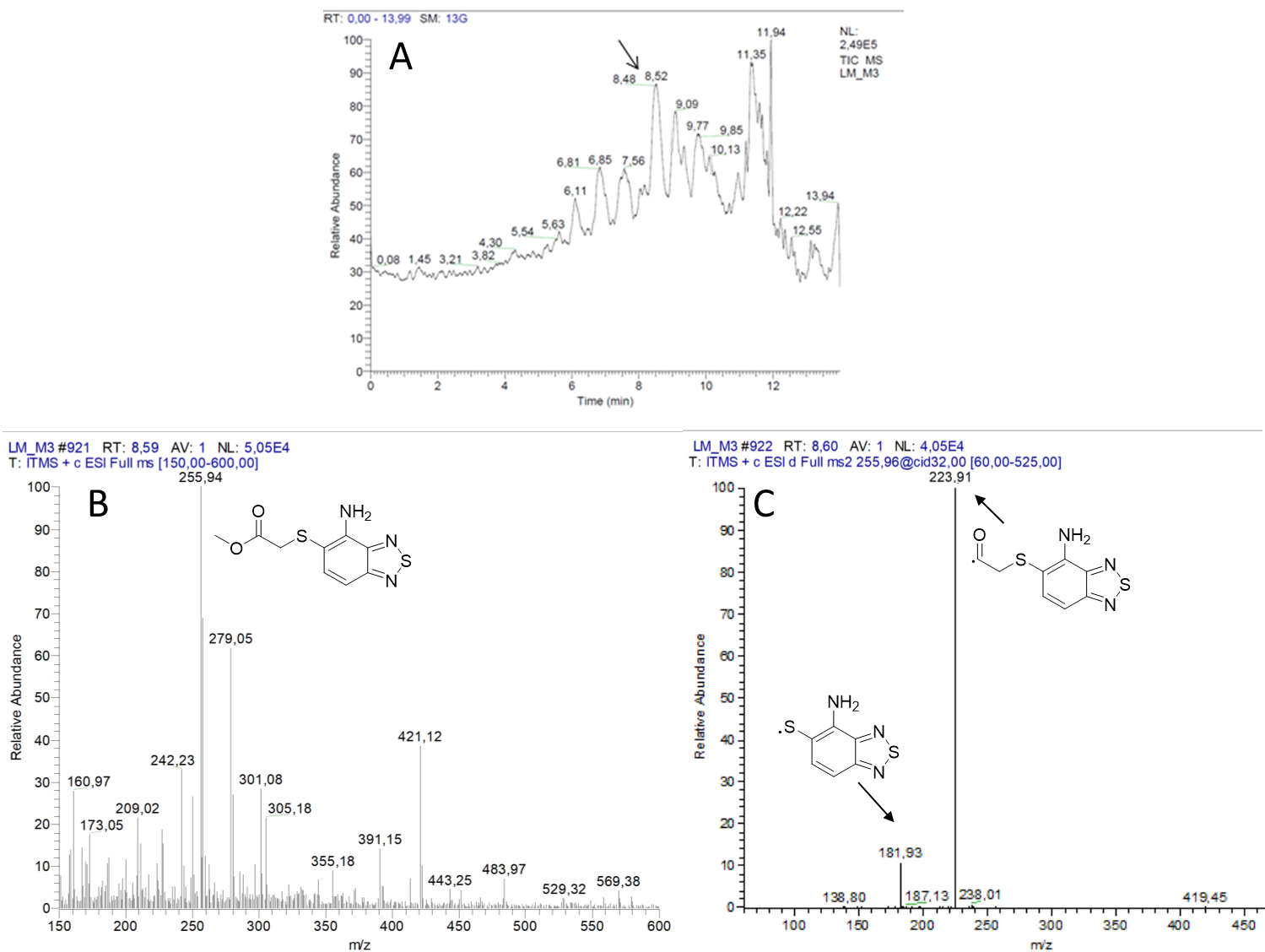


**Supplementary Figure 4.** UPLC-MS analysis of fraction M3 of the silica gel chromatography of the C109 treated *B. cenocepacia* culture extract. (A): chromatogram; (B) full-mass of the selected peak M3; (C) fragmentation pattern.


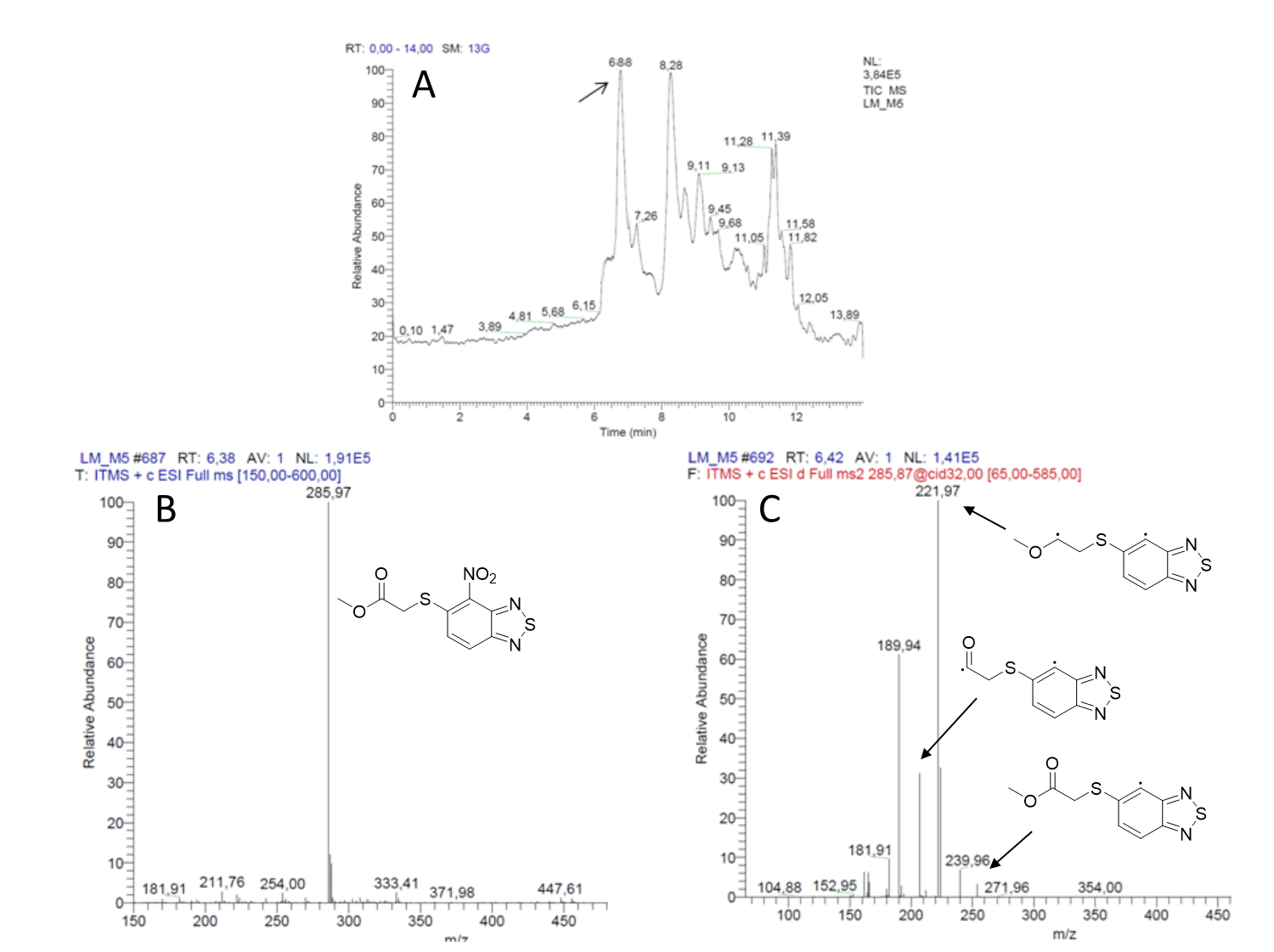


**Supplementary Figure 5.** UPLC-MS analysis of fraction M5 of the silica gel chromatography of the C109 treated *B. cenocepacia* culture extract. (A): chromatogram; (B) full-mass of the selected peak M5; (C) fragmentation pattern.


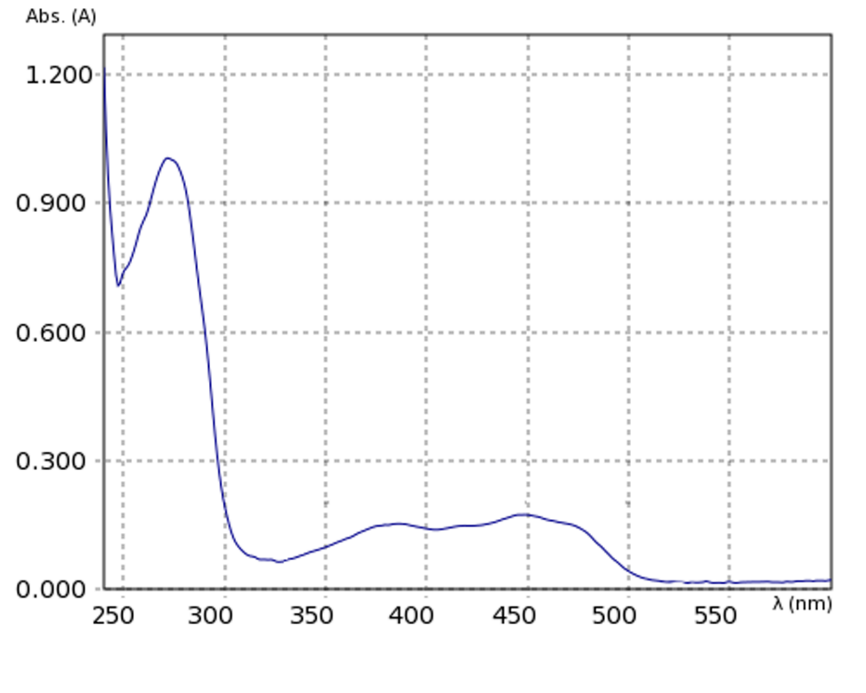


**Supplementary Figure 6.** UV-vis spectrum of the recombinant BcNR shows that the enzyme is produced as a flavoprotein.


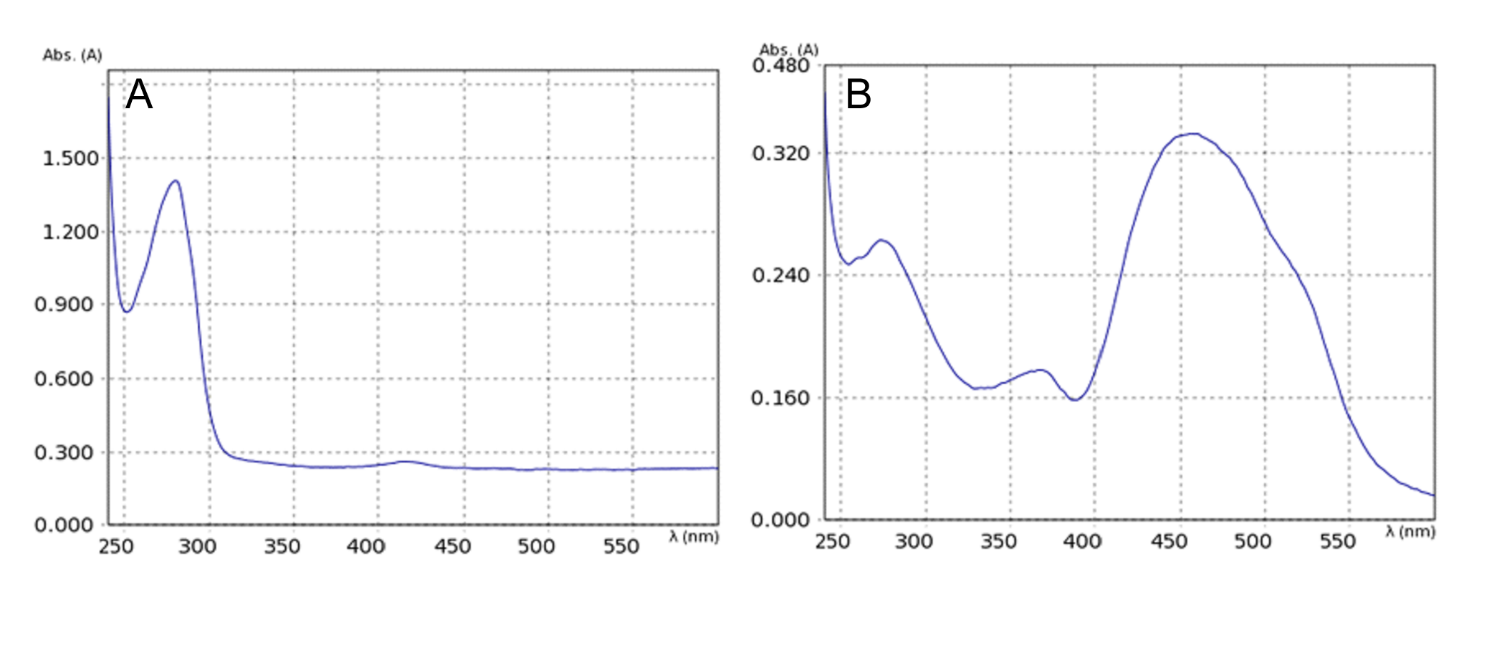


**Supplementary Figure 7.** The UV-vis spectrum of the BcNR resuspended after heat denaturation lost the peaks at 360 and 450 nm (A) characteristics of the flavin, that were found in the spectrum of the supernatant (B), demonstrating that the cofactor is not covalently bound.


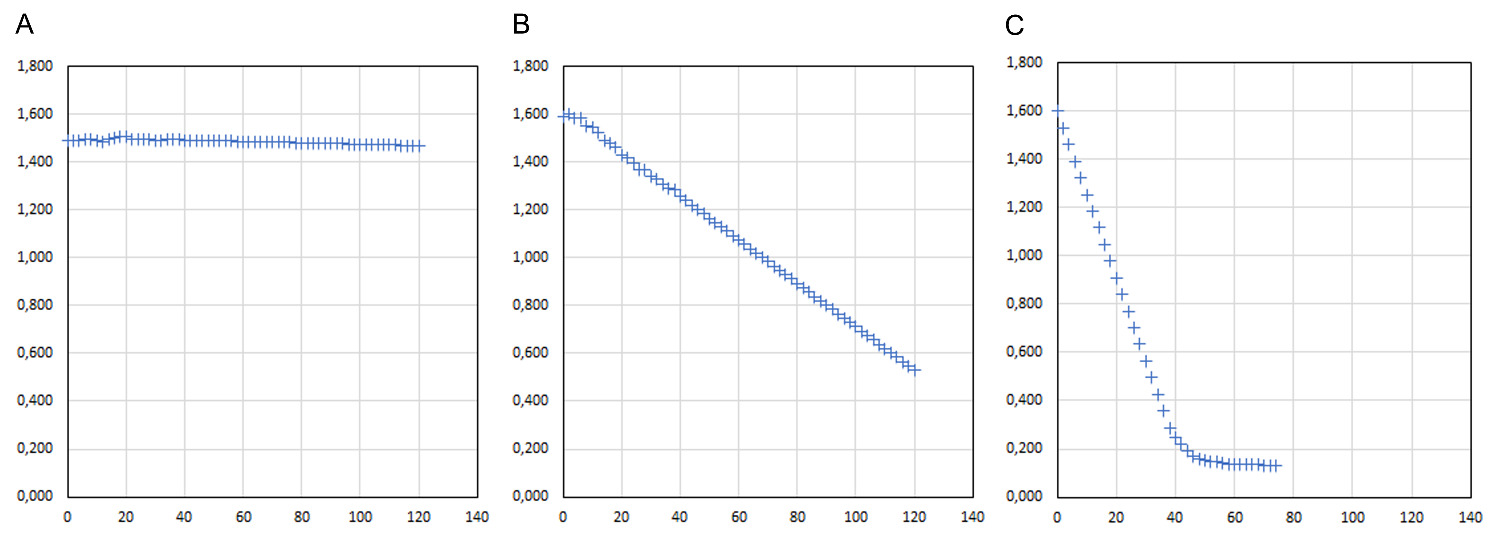


**Supplementary Figure 8**. Assessment of the enzyme assay of BcNR. The enzyme is strictly NADPH dependent, since using 4-nitrobenzoic acid as substrate, no activity was found when the cofactor was NADH (A), while in the presence of NADPH the enzyme was active (B). Moreover, the enzyme was also able to use C109 as a substrate (C).

## 2.2 Supplementary Tables

**Table S1. C109 derivatives list (uploaded as a separated excel file).**

**Table S2. Steady-state kinetic parameters of BcNR as a function of 4-nitrobenzoic acid and C109 as substrate.**

| **Substrate** | **V_max_ (U/mg)** | **k_cat_ (s^-1^)** | **K_m_ (mM)** | **k_cat_/K_m_ (s^-1^ mM^-1^)** |
| --- | --- | --- | --- | --- |
| **4-nitrobenzoic acid** | 7.6 ± 0.9 | 17.5 ± 1.4 | 0.20 ± 0.021 | 87.5 ± 8.1 |
| **C109** | 71.9 ± 2.1 | 165.1 ± 5.9 | 0.08 ± 0.005 | 2063.7 ± 20.1 |

**3 Supplementary movies**

**Movie S1. Time-Lapse Microscopy of *B. cenocepacia* J2315**.

Time-lapse microscopy of *B. cenocepacia* J2315 exponential-phase cultures. Representative movie in phase contrast in which exponential-phase bacteria were sparsely inoculated into a microfluidic device and, pre-grown in 7H9, followed by a first exposure to C109 (12.5X-MIC), drug washout and a second exposure to C109 (12.5X-MIC). Images were recorded at 20 min intervals, using a 100X objective. Time elapsed is indicated in hours. Selected snapshots from the movie are shown in Figure 6. Scale bar, 5 μm.
